# Food security and livelihoods of post-resettlement households around Kanha National Park

**DOI:** 10.1371/journal.pone.0243825

**Published:** 2020-12-28

**Authors:** Amrita Neelakantan, Ruth DeFries, Jessica Fanzo

**Affiliations:** 1 Department of Ecology, Evolution and Environmental Biology, Columbia University, New York, New York, United States of America; 2 Department of International Health of the Bloomberg School of Public Health at Johns Hopkins University, Baltimore, Maryland, United Stated of America; Sveriges landbruksuniversitet - Campus Umea, SWEDEN

## Abstract

Globally, conservation efforts have moved millions of people out of protected areas since the 1970s, yet quantitative studies on post-resettlement well-being remain a challenge due to poor documentation. Since 2008, the Indian forest department records demographic and financial details at the household level under standardized guidelines for resettlement. Here, we examine the food security of approximately 600 households’ post-resettlement from Kanha National Park (KNP) in central India between 2009 and 2014. We compare food security of resettled households with host community households with a total of 3519 household surveys, conducted over three seasons within one year. We measure food security using food consumption scores (FCSs), coping strategies index (CSI) and household hunger scale (HHS). Food insecurity is widespread in the landscape, with over 80% of households reporting poor or borderline FCSs year-round. Additionally, we recorded food insecurity increases in monsoon for all households regardless of resettlement status. Results indicate that resettled households are comparable to their host community neighbors in FCS and all households use mild coping strategies to combat food insecurity. While widespread, food insecurity in the KNP landscape is not acute with very few (<10) reports of severe hunger (as measured by the HHS). Almost all foods are market bought (>90%) and sometimes supplemented by gathering locally prevalent greens or from kitchen gardens (forest dependency for food was negligible). Accruing assets and diversifying incomes from non-labor avenues would alleviate food insecurity for all households. The patterns of market dependence and food security associated with diversified stable incomes around protected areas is in contrast with many studies but is likely to occur in similar human-dominated landscapes.

## Introduction

Conservation-related resettlements trace back to the establishment of Yellowstone National Park in 1872 and spread rapidly with fortress management policies to conserve endangered species habitats. Globally recognized areas of high biodiversity are inhabited by more than a billion people and populations in these regions continue to grow at a rapid pace [1, 2]. Protected areas cover more than 12% of the earth’s terrestrial surface, a recognized conservation success, but have displaced millions of people from these areas [3–5]. In India, the Society for Participatory Research in Asia (PRIA [6]) estimates that the establishment of protected areas displaced more than 600,000 tribal people. Conservation practice, from origin to present day, has often been at odds with the well-being of local human communities [7–9]. Conservation related resettlement has been widely criticized as an extension of neoliberal policies with negative consequences for local communities’ rights and ownership of biodiverse areas [10, 11]. Researchers continue to have concerns about the nature of voluntary resettlement in conservation practice and that conservation remains top-down in decision making with few opportunities to include local communities as equal stakeholders [12–14]. Empirical research on the well-being of displaced people constitutes a small but growing literature, one that was historically challenged by small samples sizes and non-comparable methods [15–17]. Quantifying impacts within the historically polarized debates between human development and protected area management can aid future management for people and wildlife [18, 19]. Even with decades of efforts to reconcile protected area management with human development, studies of the trade-offs and synergies between biodiversity conservation and the well-being of people often find conflicting and complicated results usually explained by local context [20, 21]. Researchers now consider wildlife conservation from a systems perspective to achieve the twin goals of human development and conservation [22, 23].

Understanding the impacts of conservation-related resettlement on people requires meaningful measures of human well-being. Income based metrics of well-being are unidimensional and do not reflect livelihoods reliant on goods with little economic value (for example–wild foods or self-provisioning via non-economic landscape resources) [24, 25]. More composite metrics such as the Quality Of Life measurements or the Human Development Index include more dimensions, but are difficult to interpret [26–28]. Food security has recently gained favor as a measureable and multidimensional metric of human well-being by tying together livelihoods and natural resource use [29–31]. Measures of food security and livelihoods provide a means to understand the impacts of human resettlement from protected areas [32, 33]. Interdisciplinary case studies provide vital local inferences to explore social-ecological frameworks in practice [15, 34–36].

In India, the current resettlement policy is explicit in its goal to resettle people to expand and maintain critical tiger habitats within 50 protected areas (Tiger Reserves) with detailed resettlement records and standardized compensations [37]. Resettlement has been and remains a contentious political reality while studies often suggest, but do not conclusively prove, that big cats and human populations cannot continue to coexist within India’s protected areas [16]. The standardized monetary compensations allow households to move to locations where they purchase land (as opposed to previous schemes in which park managers displaced households to specific locations). The current resettlement provides an opportunity for a data rich study with potential for replicability across the country. Most importantly, India is a megadiverse and high human density country which mirrors the socio-economic and conservation realities faced by many nations with intense competition for natural resources [38, 39]. Kanha National Park, recognized internationally for tiger tourism, is important for tiger dispersal allowing for healthy genetic diversity in the central Indian populations [40]. The government of India recognized the efforts of the KNP’s forest department with an award for their documentation and implementation of the current resettlement policy.

We explore food security at resettled households compared to their neighbors at their new settlement locations across the Kanha National Park landscape to answer the following questions:

1. Are resettled households moving into remote areas compared to all existing villages in the study site, specifically with respect to–road access, food markets and forest availability?
2. Do resettled households have comparable Food Consumption Scores (FCSs) and Coping Strategy Index (CSI) measurements to their host community neighbors? (See–methods and S2 File for more information on food security metrics)
3. What livelihood characteristics are associated with FCSs in the KNP landscape? Do these differ for resettled households and their host communities?

## Methods

### Study site

**Fig 1:**
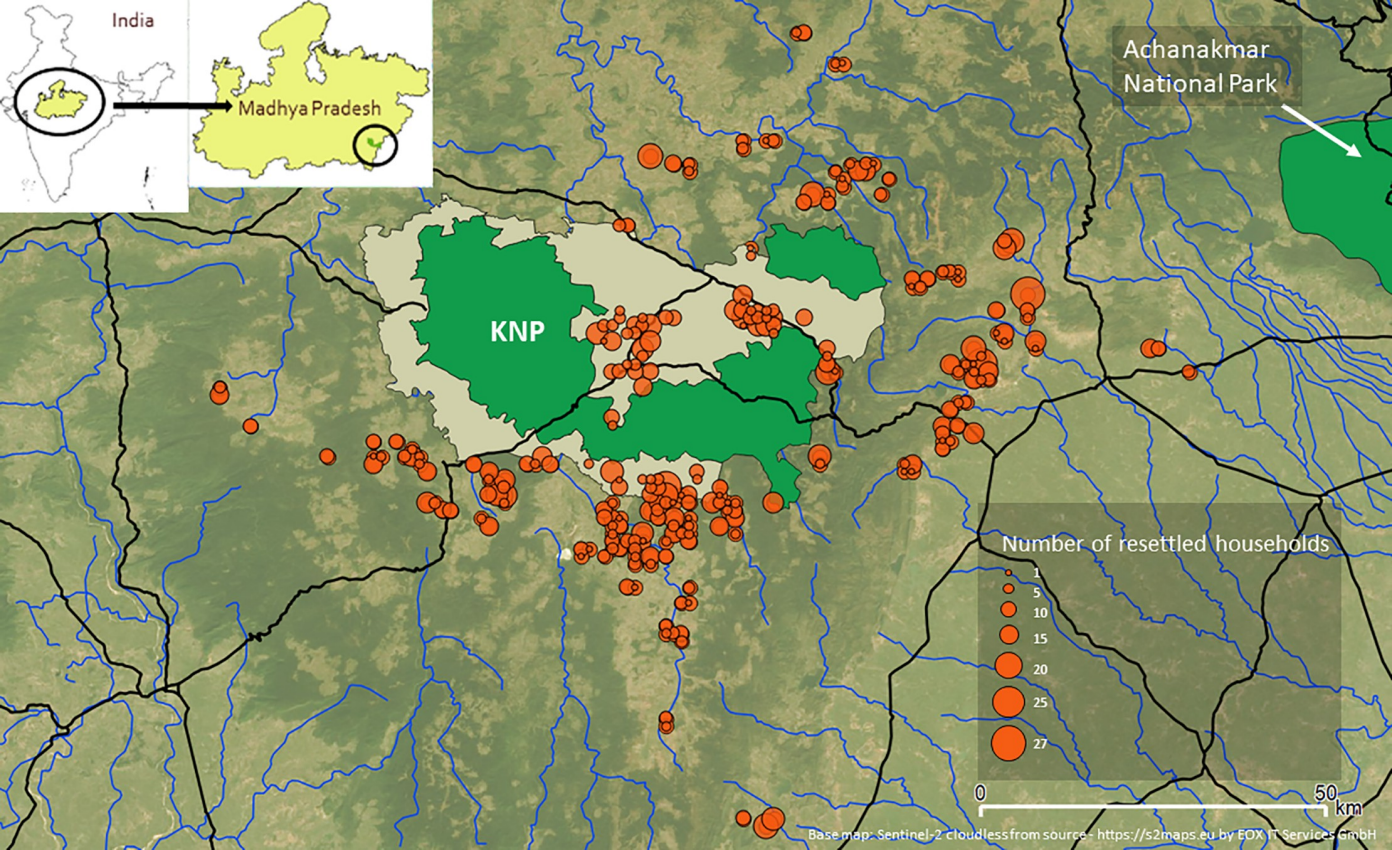
Study site and surveys of households. KNP has a core (green polygon within beige buffer) and Phen Wildlife Sanctuary as a satellite core (green polygon) in the north is adjoining the multi-use KNP buffer (beige). The non-protected forest corridors join protected areas and lead out of KNP, boundary of Achanakmar National Park shown as incomplete green polygon in top right corner of map. Orange circles indicate number of resettled households from KNP at their new settlement locations. Black and blue lines respectively indicate roads and rivers. Basemap is Sentinel-2 cloudless from source - https://s2maps.eu by EOX IT Services GmbH (Contains modified Copernicus Sentinel data 2016 & 2017). Inset maps show location of KNP in Madhya Pradesh state in central India.

Kanha National Park (KNP) (22.3333° N, 80.6333° E), established in 1955, is one of India’s most well-known Tiger Reserves. KNP is approximately 940 km^2^ in area, with a multi-use buffer of approximately 1005 km^2^. Mixed deciduous forests and grasslands form the typical vegetation and support populations of tiger (*Panthera tigris*), leopard (*Panthera pardus*), wild dog (*Cuon alpinus*), sambar (*Cervus unicolor*), chital (*Cervus axis*), barasingha (*Cervus duvaucelii*) and gaur (*Bos gaurus*) [39] (Fig 1). Human population densities range between 182–195/km^2^, and livestock densities range between 65–79/km^2^ in the districts adjoining park boundaries [39]. The central Indian region’s human population includes a high density (>25%) of historically disadvantaged indigenous “scheduled tribes” or *adivasis* [41]. Villages across the KNP landscape, within the park buffer and outside, reflect the high proportions of tribal populations found in central and north-eastern India [42]. Most households are agrarian and purchase food from local weekly markets. Agrarian households in this landscape are heavily dependent on the monsoon season as their main growing season and most irrigation is rain fed. The seasonal differences in livelihood strategies are likely to affect all aspects of rural living including food security. In the study region there are three seasons annually–summer (April—June), monsoon (July—September) and winter (October–March). Summers are hot and dry, monsoons are largely responsible for the annual precipitation with winters being cool and dry. Human livelihoods around the protected area rely on seasonal commercial and subsistence forest goods, including a heavy year-round reliance on forests for fuelwood (nearly 100% in this area compared to the 77% national average) and cattle grazing [43].

As in many other protected areas, the KNP management displaced villages, to conserve habitats within protected areas, in the 1970s and then again in 1980s with compensations of land and financial aid [44]. The National Tiger Conservation Authority (NTCA) approved a standardized protocol in 2006 that follows guidelines found in the National Resettlement and Rehabilitation policy (2007) to create inviolate areas for tiger conservation [37]. The National Resettlement and Rehabilitation policy (2007) provides government mandated processes from the date of announcement until it is either amended or dissolved. This policy includes voluntary resettlement of households with monetary compensation, an option that is most common in practice in KNP. The Indian Forest Department uses the NTCA guidelines for resettlement and records demographic and financial details at the household level of all resettled households (2008 onward). The KNP managers followed the guidelines as per the Government of India and resettled households opted for the first option in the guidelines “In case, the villagers through the Gram Sabha agree for receiving a payment of Rs. 10 lakhs per family (inclusive of the valuation for their assets), then the said amount would be deposited in the name of the beneficiary (a joint account with spouse in case of a married individual)” [37]. The current policy is in contrast to historical evictions in the 1970s and 80s from KNP in that it is not directed, provides standardized monetary compensations and households can move to areas in the landscape where they can procure land. Resettlement remains a contentious conservation policy due to implementation of conflicting goals–to create inviolate habitats for wildlife while upholding the legal rights of human communities within protected areas [8, 12, 45]. The NTCA policy highlights changes in resettlement policy in response to criticisms of historical evictions from protected areas. From 2009 onwards, managers of KNP resettled approximately 850 households under the NTCA 2006 resettlement protocol. Not all households resettled at the same time—with some leaving in 2009, others as recently as 2013. We consider only the resettled households from 2009 onwards to minimize the effects of unknown confounding factors from the previous resettlement programs of the 70s and 80s.

### Scope of study

We quantitatively address outcomes of resettlement for human well-being using food security as a tractable and multidimensional metric. We explore patterns of food security across the landscape by surveying 1173 households three times in one year (2015) (Columbia University IRB, protocol number AAAN5603, exemption subsection 45CFR46). Our research cannot compare households inside (pre-resettlement) to those who have moved outside (post-resettlement) of the park as no households remained inside KNP core at the time of study period or thereafter. The current National Tiger Conservation Authority (NTCA) policy for resettling households is clear that “The ongoing study and the analysis of the available research data on tiger ecology indicate that the minimum population of tigresses in breeding age, which are needed to maintain a viable population of 80–100 tigers (in and around core) require an inviolate space of 800–1000 sq. km. Tiger being an “umbrella species”, this will also ensure viable populations of other wild animals (co-predators, prey) and forest, thereby ensuring the ecological viability of the entire area / habitat. Thus, it becomes an ecological imperative to keep the core areas of tiger reserves inviolate for the survival of source populations of tiger and other wild animals” [37]. Moreover, as a reversal of the policy is not likely, research can most usefully contribute through improved understanding of post-resettlement impacts. We focus our analyses on household food access measurements, but provide information on pertinent landscape wide features that underpin food access–for example weekly markets, forest cover and kitchen gardens.

### Sampling of host community households

We surveyed host community households at the villages where resettled households are now located to form a useful and appropriate baseline in our comparative analyses. We define host community as the existing community within a village that resettled households have joined since leaving KNP. These villages are already established villages. Such an approach has found favor in comparisons when true controls are difficult to survey due to circumstantial (all households resettled into non-comparable populations and none remained within the park) or logistical difficulties (no access to resident households within parks that still remain) [46, 47]. The field assistants sampled every seventh house in a random walk method using a coin toss to change direction (left or right turns) [48, 49]. This was necessarily flexible to be able to sample host community households in villages of different shapes and sizes. The number of such selected host community households in each village follows the distribution of resettled households across the survey villages. In the case of one or two resettled households in a village, we surveyed three or five host community households to ensure a sensible baseline. We surveyed the same households each season for resettled and host community households, unless we could not locate household members in a given season. In the case of household members not being available for survey in a resettled household, we omitted it from our seasonal survey protocol. Host community neighbors at the new settlement villages provide a baseline to compare food security measurements and livelihood characteristics. We clarify that unless explicitly mentioned we use households to signify all our surveyed households regardless of resettlement status.

### Sampling design and total survey effort

We surveyed 578 resettled households from KNP under the current resettlement policy and 595 host community neighbors. The KNP forest department provided us data to locate all 850 of the resettled households at their new settlement locations across more than 157 villages across five districts of the two Indian states–Madhya Pradesh and Chhattisgarh. Within districts there are administrative units called *tehsils* that we use in our study to more accurately draw comparisons between resettled households and local populations. In the case of household members not being available for survey in a host community household, we randomly sampled another household (n = 224 in monsoon season). Our seasonal drop-out rates for host community households (from n = 702 in summer, to n = 513 in monsoon, surveyed households in winter n = 571) occur across villages with more than three or more resettled households. The drop in the number of resettled households in our surveys (from 850 to 578) is predominantly due to households merging when resettling outside of the park as well as a few households (<10%) that we could not locate. The merging of households largely pertains to definitions of what constitutes a single household, the interpretation of the resettlement guidelines led to single household compensations for all adults within a family that continued to cohabit post-resettlement in a single physical home [37].

We carried out surveys in three seasons to capture seasonal dynamics of food security and livelihood characteristics in summer, monsoon and winter (May 2016 –January 2017). We consider livelihood characteristics to include the choices households make (for example–what activities to undertake to diversify incomes, increase number of cattle or buy a motorcycle, among others). To measure the remoteness of survey locations we calculated forest cover per available capita, distance to—built-up area, highway, closest market, river and the core forest of Kanha National Park. Rivers were included in our measure of remoteness in the case economic activities and facilities were reaching areas with means for all-year irrigation, as compared to remote locations with only rain-fed agriculture. Built-up areas is a point layer across India [50]. We conducted semi-structured interviews, at each survey household, to collect data on socio-economic status, food access measurements and interactions with forests. In total, we conducted 1332 surveys in summer (630 resettled, 702 host community), 1066 surveys in monsoon (553 resettled, 513 host community) and 1121 surveys in winter (550 resettled, 571 host community). To conduct this extensive survey effort we employed 12 to 14 field assistants each season for approximately three to four weeks. The same survey team collected data and training was refreshed before each field season. Additionally, the first author of the study oversaw the field team throughout the three field seasons. Any surveyor biases remaining due to personal differences would be uniformly spread across our surveys due to sampling design and field protocol of mixing surveyor teams and locations.

### Food security metrics

**Fig 2:**
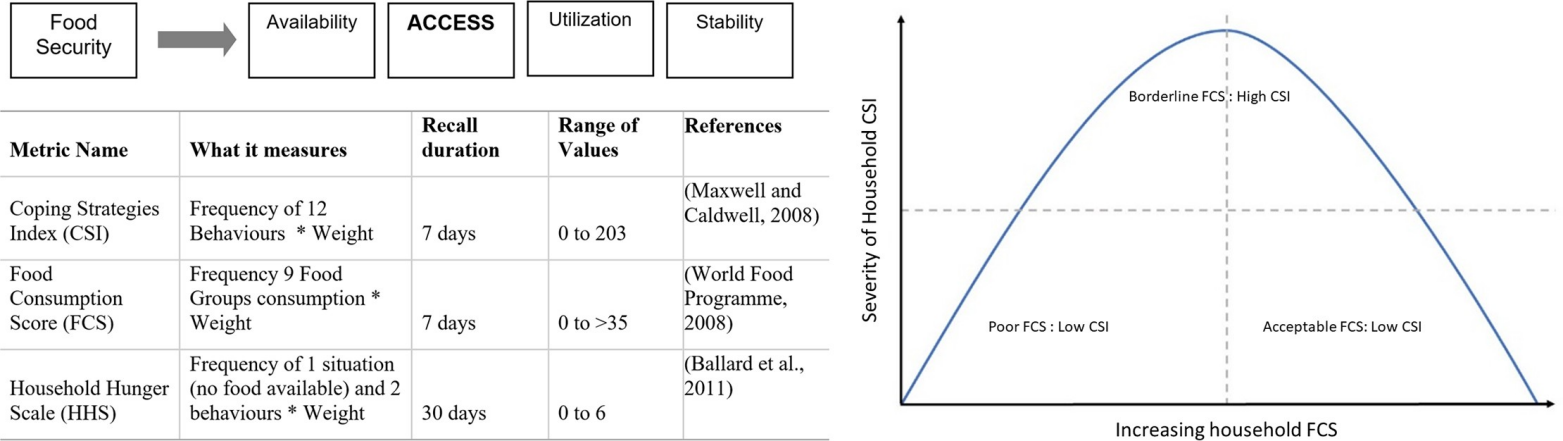
Metrics for food security assessments. (a) Diagram shows the four pillars of food security. We used three standardized metrics to explore household access to foods available at their village location [53, 56–58]. For more details please see S2 File. (b) Hypothesized relationship of FCS and CSI—CSI will be positively correlated with FCSs until households attain higher FCS in borderline category or acceptable FCSs. After attaining higher FCSs we expect to see a negative association with CSI.

We define food security in our study according to the four pillars of food security: availability, access, utilization and stability (Fig 2A) [51]. We use validated standardized multi-dimensional metrics that are scale appropriate (measured at the household level) with appropriate recall time to assess food security in the KNP landscape [30, 52]. To measure availability we measured produce and prices in markets across the landscape as well as asked about forest foods in semi-structured interviews. We used the Coping Strategies Index (CSI), the Food Consumption Score (FCS) and the Household Hunger Scale (HHS) to measure access (or lack thereof) to foods at the household level [53] (Fig 2A and S2 File). Finally, to measure stability we surveyed households three times in a year to capture seasonal variation and recorded incidence of shocks to the households. The field teams surveyed markets to gather baseline data on location-specific food availability with prices from two vendors wherever possible. Market data is key to discern the physical availability of foods across the landscape (S5 File). We focused on quantifying food access using metrics of diet diversity and the behaviours associated with coping in times of food scarcity. We acknowledge that our measurements focus on the outcomes of household access resulting in recorded FCSs, CSI and HHS scores instead of direct measurement of the many aspects of accessing resources [54, 55]. Instead, we use response on some of these aspects of accessing resources in our models explaining patterns of FCSs.

The FCS and CSI are weighted scores based on the food groups consumed and coping behaviors exhibited in the last seven days in the surveyed household respectively [56, 57]. We use the cross-culturally validated Household Hunger Scale (HHS) in our study to categorize households with—‘little or no hunger’ (0–1), ‘moderate hunger’ (2–3) or ‘severe hunger’ (4–6). The HHS is calculated by recording the frequencies of severe behaviors in times of food scarcity, recalled over 30 days prior to the survey [58]. Categorization of the coping strategies as mild, moderate and severe are assigned with higher weights attached to more severe coping behaviors, the behaviors listed from mild to severe were validated in focus groups in the KNP landscape before undertaking the surveys (S2 File). For example, eating less preferred or cheaper foods was considered a mild coping behavior in comparison to severe behaviors such as—skipping meals or going a day without any meals. The FCS scores are similarly weighted scores classified as poor FCS, borderline FCS and acceptable FCS. The FCS and CSI can together provide information on food quality and access, but the HHS provides more accurate measures of high levels of food insecurity [58]. CSI in addition to FCS data can shed light on whether households are being able to cope (CSI positively correlated with FCS) in times of scarcity or if households are struggling to source foods (CSI negatively correlated with FCS) (Fig 2B). We hypothesize that in rural India the relationship of FCS and CSI will be such that CSI will be positively correlated with FCSs until households attain higher FCS in borderline category or acceptable FCSs. After attaining higher FCSs we expect to see a negative association with CSI (Fig 2B). For more details on focus groups that informed our choice and use of food access metrics please see supporting information of this study (S2 File).

### Data analyses

We used propensity scoring and visual inspection (of the overlap between resettled households and host community households) for all measured variables to test balance in our study sample, especially to ensure that our sampled host community households provided a comparable baseline for surveyed resettled households (S1 File). Additionally, because all households that were previously within KNP have moved out of KNP, no characteristics make a household more or less prone to resettlement. We also explored if our surveyed villages were comparable to other villages found in the geographic extent of the study. We did this by identifying all villages within the convex hull of our resettlement point locations and then ran a PCA using variables of remoteness and village level assets (proxy for economic opportunities) for all villages and our surveyed villages (S4 File). We included a PCA to see overlap of remoteness, village size and forest land between our survey location compared to all villages in the study site to understand if resettled households had picked isolated remote locations within the study area.

In the KNP landscape, households buy foods from weekly markets and therefore restock each week for fresh vegetables, meats and staples. For availability of market items and their prices, we visually compared market checklists as most markets in the landscape have the same produce with similar prices (S5 File). We also asked respondents about consumption of forest foods, kitchen garden produce and measured forest available per capita at each new settlement location to measure supplementary sources for local food baskets. The weekly cycle of accessing markets in the landscape also influenced our choice of metric to measure diets at households–we deliberately chose the food consumption score (FCS) as it too has weekly cycle of recall. We compared means of food access metrics (FCS, CSI and HHS) to ascertain if resettled households were food secure in comparison to their host community neighbors. To understand if resettled households and host community households had similar access to food we compared them using standardized thresholds for adequate, borderline and poor FCS [56]. We used t-tests, ANOVAs and the Tukey HSD post-hoc tests to find significant differences in food access between resettled and host community households. We also modelled socio-economic data collected as each household to explain our overall distributions of FCSs in which CSIs were a determinant variable. We clarify that our analyses cannot explain causal mechanisms of livelihood choices leading to food insecurity or predict the future food security status of a household.

We used Euclidean nearest neighbor distances to find distances between household locations and nearest road, border of protected area (KNP core), market and built up area. We used existing GIS layers of roads, protected area borders, built up area and our GPS locations of markets [50, 59]. We also created buffers of four kilometers around each household GPS point and calculated forest cover within the buffer as number of pixels of forest cover [60], because most people access nearby forests routinely for fuelwood and for grazing cattle. Cattle can be grazed further away but fuelwood is typically collected within a daily walking distance of a few kilometers region-wide [61].

To understand patterns and associations in our household level data we used cluster analyses (ClustVis PCA–see [62]), random forests and mixed effect models. We explore clustering in our data with all variables used in subsequent models. We used random forests and mixed effect models to understand if resettled and host community households have differing livelihood characteristics associated with food access measurements (S9 and S10 Files). We used mixed effect models to evaluate important variables, identified in random forest analyses, to understand linear associations with FCSs resettled and host community households separately. We included two random effects in our models for local administration (*tehsil*—for all households) and origin village (only when modelling food access in resettled households) (S8 File). We standardized all determinant variables used in mixed effect models to ensure that the unit of measurement does not have an impact on our results, and their consequential interpretation. In our comparisons of means, we did not transform or standardize our data. We conducted all statistical analyses using the software R (http://www.r-project.org) and QGIS 2.10.0- Pisa (Quantum GIS Development Team, 2015, qgis.os-geo.org). Data is available at (https://bit.ly/2GvxGid).

## Results

### Are resettled households moving into the remote areas compared to all existing villages in the study site with respect to–road access, food markets and forest availability?

**Fig 3:**
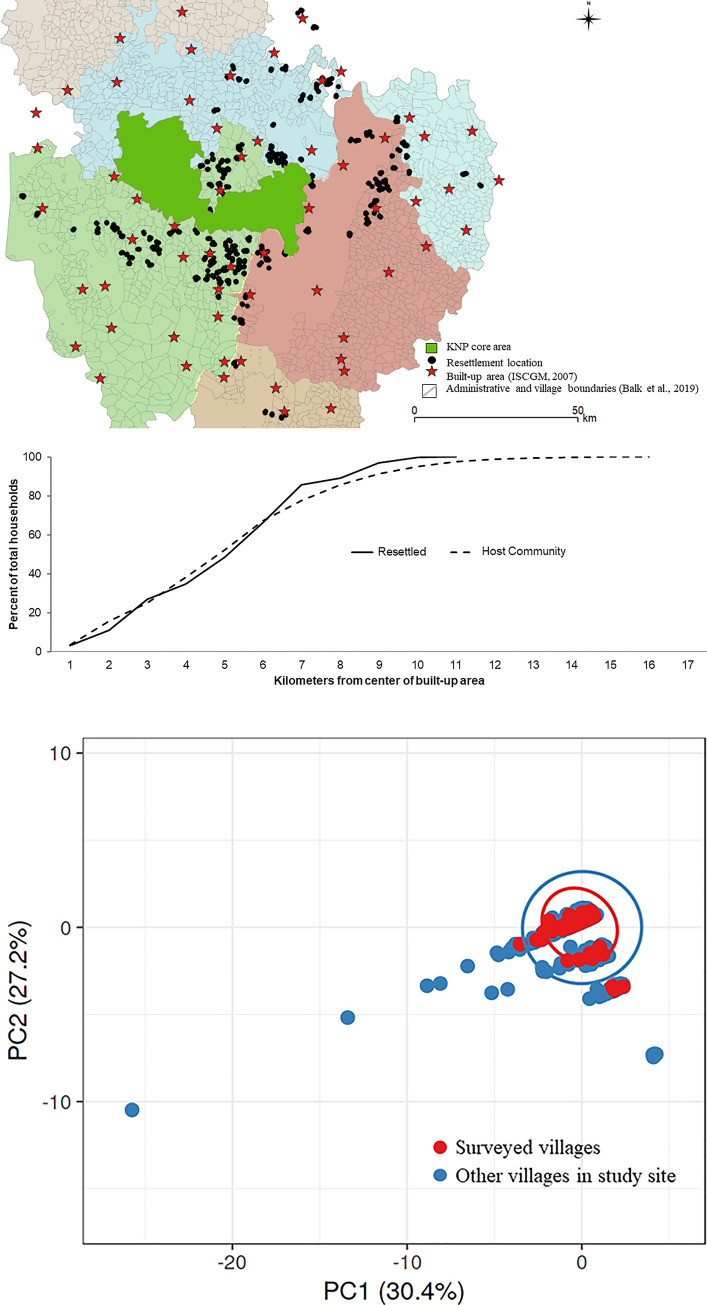
Survey is a subset of landscape-wide characteristics. (a) Map shows locations of built up area (red stars) [50], village boundaries color coded into administrative units (*tehsils*) [64] and locations of resettled households (black dots) used to calculate Euclidean distances between rural populations and urban centers. (b) Resettled households proportionally mirror densities of host community human densities across urban-rural transition in the KNP landscape (chart below map). In our study, resettled households were within 11 kilometers of a built up area (for other remoteness measurements see S3 File). (c) PCA results showing that our surveyed villages (red) are clearly a subset of the larger set of villages (blue) in the landscape–when compared across variables of village area (including forest area and unirrigated area) and remoteness measurements (presence of highways and distance to nearest town) using data from the Government of India census [43]. X and Y axis show principal component 1 and principal component 2 that explain 30.4% and 27.2% of the total variance, respectively. Prediction ellipses are such that with probability 0.95, a new observation from the same group will fall inside the ellipse. N = 1076 data points (for more PCA details and density plots of variables used see S4 File).

Resettled households have predominantly moved into existing villages in similar proportions to host community human densities across the KNP landscape (Fig 3). However, in absolute numbers, resettled households make up a negligible fraction of the households already residing in their host communities [63]. Out of 850 households resettled, only four left the landscape to two cities (Bhopal and Durg). Approximately 20% of surveyed households in our study are located within the KNP buffer (Fig 1). Resettled households moved out individually, not as a whole village. The resettled households join existing villages and most remain close to KNP (90% of surveyed households within 30 kilometers of KNP core boundary) in comparison to all existing villages spread across the study site (S3 File). In the study site, all households have access to forest (mean 506 sq. meters within a 4 sq. kilometers) and are within 11 kilometers of a built-up area (S3 File).

Households in the KNP landscape typically access the bulk of their foods (>90%) from weekly markets at their village or a neighboring village (See methods and S1 File). Thus, resettled and host community households have similar availability of food across the study region with physical access to already established weekly markets.

### Do resettled households have comparable FCSs and CSIs to their host community neighbors?

**Fig 4:**
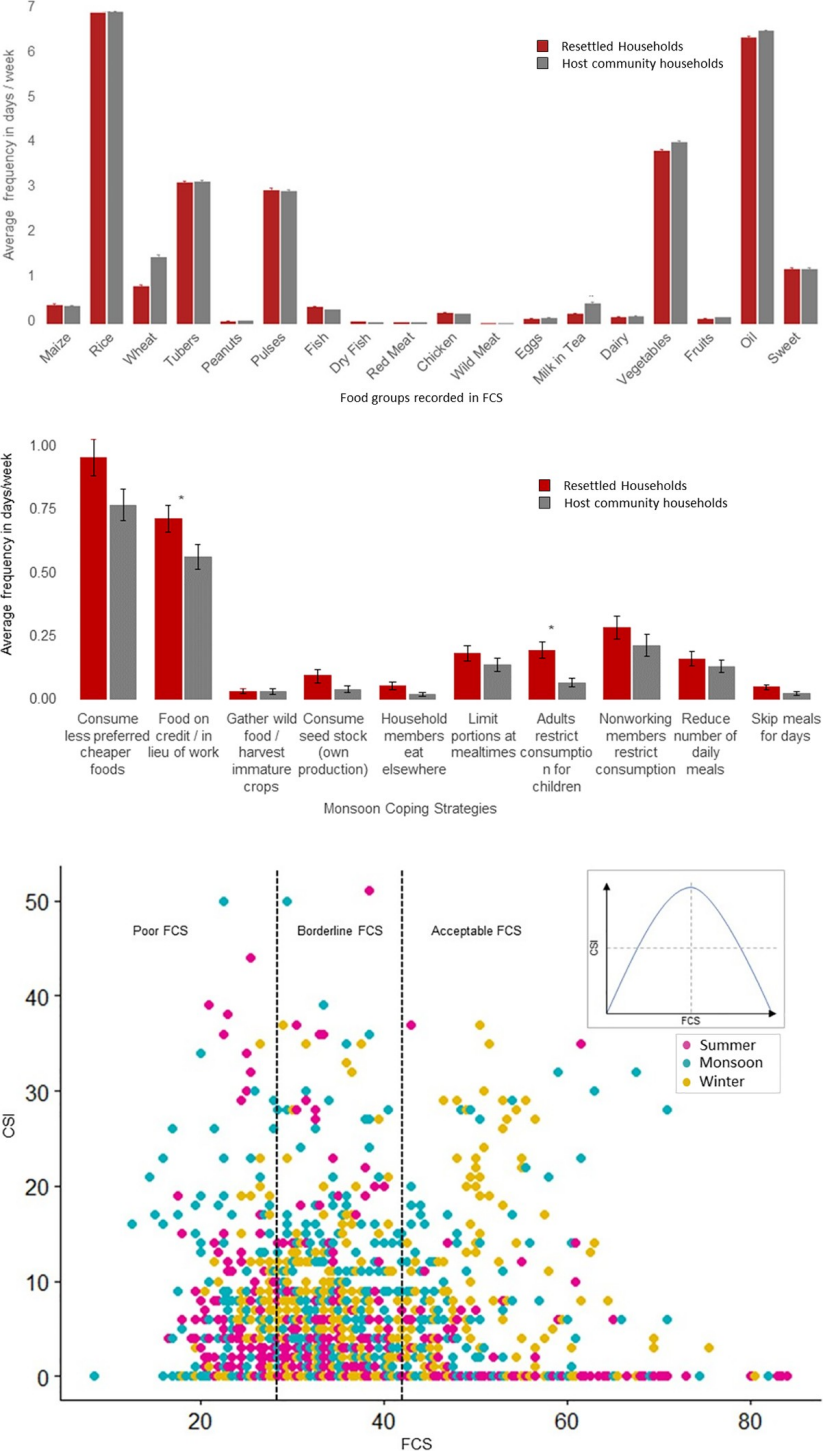
Comparisons of FCSs (a) and monsoon CSIs (b) between resettled and host community surveyed households. (a) Resettled (red) and host community households (grey) show similar frequencies of food groups consumed. (b) Resettled (red) and host community (grey) household employ all coping strategies in monsoon, but resettled households use all of these coping strategies more frequently than their host community neighbors. * indicates p<0.05 in t-test comparisons. (c) CSI and FCS of all households across seasons (summer–pink, monsoon–blue, winter–yellow) show that CSI are positively correlated with FCSs in households with less than acceptable FCSs.

Resettled households reported similar Food Consumption Scores when compared to their host community neighbors across all three survey seasons (Fig 4). The year round average FCS for resettled households was 34.49 and for host community households was 34.36 (p-value = 0.68). In our study, households consume food groups in similar frequencies with cereals, tubers and pulses making up more than 65% of the total FCSs across seasons and locations (Fig 4). In Baihar *tehsil* during summer, resettled households (mean FCS = 36.24, n = 122) had higher mean FCSs compared to host community households (mean FCS = 31.13, n = 147) (p-value = 0.001). However, across all surveyed households with acceptable FCSs, vegetables were not consumed every day and meats very occasionally.

Resettled households had higher CSIs in monsoon across two administrative units (Baihar and Panderia) and higher CSIs than host community households in winter in Baihar and in summer in Panderia (S7 File). Moreover, the higher monsoon CSIs in resettled households are due to higher frequencies of all coping behaviors employed by all households (Fig 4). No households reported very high absolute values of CSI and used severe coping strategies rarely. The predominant coping strategies are “consuming less preferred foods” and accessing “food in lieu of work or on credit” regardless of resettlement status. In conjunction with the FCS, the household CSI is correlated with gaining access to foods in households with less than acceptable FCSs (Fig 4C) as hypothesized in Fig 2B.

**Fig 5:**
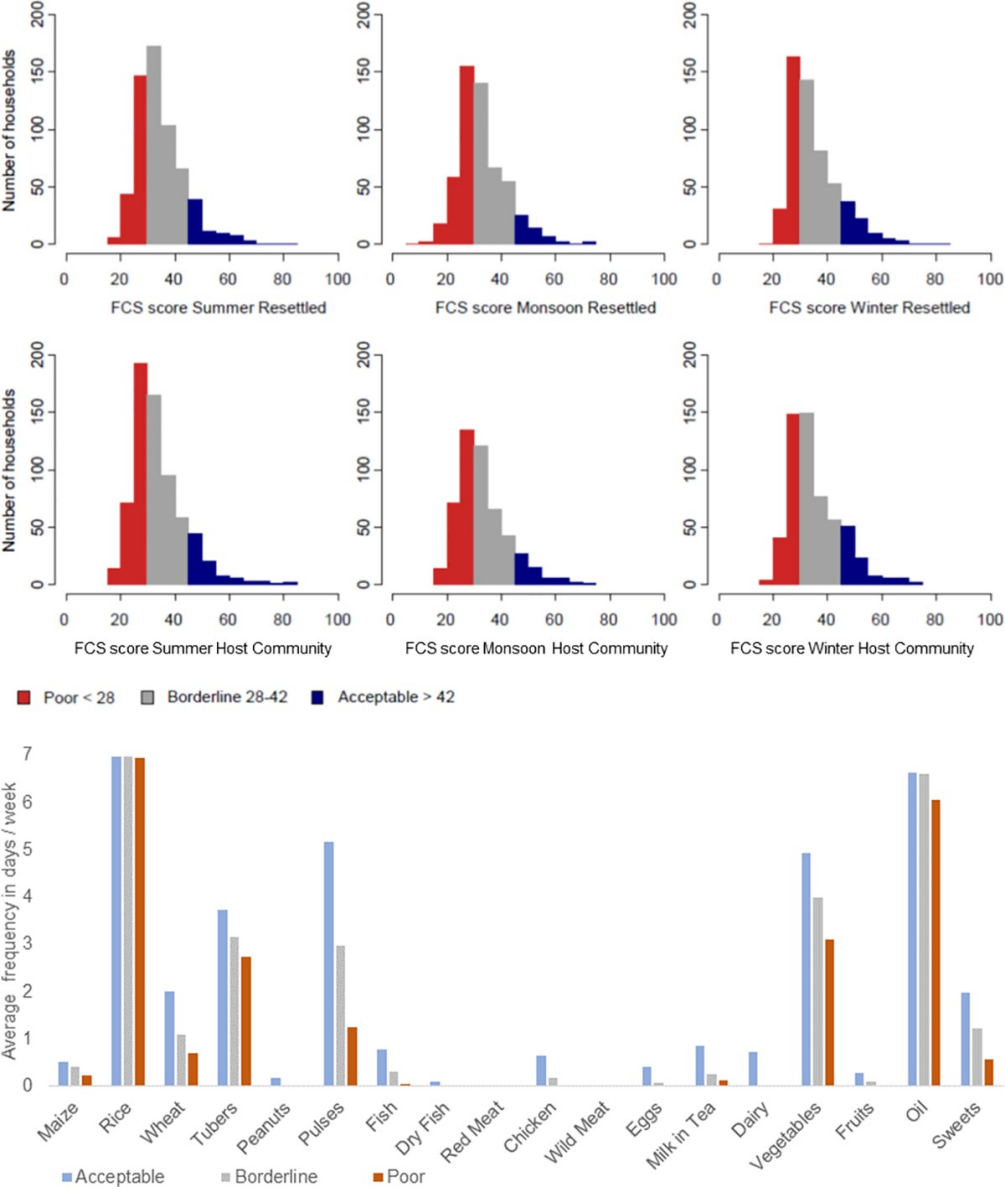
Overall, resettled and host community households (a) have the similar proportions of poor, borderline and acceptable food consumption scores. Households with poor (orange) or borderline (grey) FCSs reported diets dominated by staples (b) and with lower values of other food groups (b).

While resettled households consume food groups in similar frequencies to host community households, we find that approximately 80% of all households have inadequate levels of food consumption (borderline or poor) (Fig 5A). Resettled households and host community households have the same proportions of households being able to access acceptable, borderline and poor levels of FCSs. The number of households reporting poor FCSs rises to approximately 30% in monsoon (Fig 5A). Poor FCS households predominantly consume lower frequencies of non-staple food groups (vegetables and meats) (Fig 5B).

All households predominantly access food through market purchases (>90%) supplemented by self-provisioning (from landscape resources–non-economical and non-forest stores for foods such as communal food lots, gathering local plants from village areas and kitchen gardens) year round (S6 File). Only a handful of households forage for vegetables (1–7 households across seasons) and hunt (2 households in monsoon) (S6 File). Across seasons and resettlement status, we recorded only three instances of severe hunger (one at a resettled household) and only 94 out of all 3519 household surveys reported moderate signs of hunger (51 resettled, 43 host community) using the HHS.

### What livelihood characteristics are associated with FCSs in the KNP landscape? Do these differ for resettled households and their host communities?

**Fig 6:**
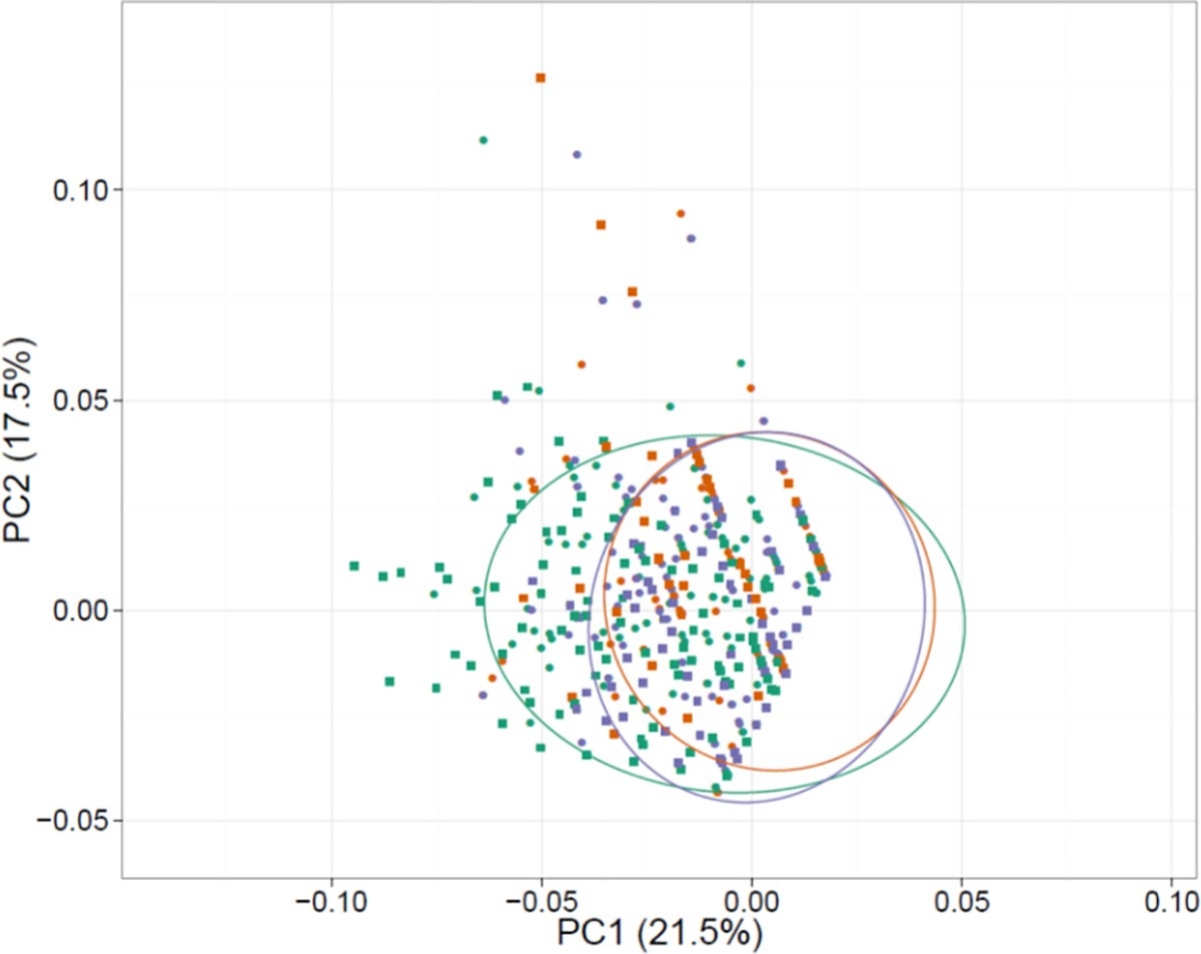
PCA results with the two most explanatory components as x and y axes showing similarities in livelihoods (variables used in models–see Table 1) of resettled and host community households. Colors signify seasons–Summer (red), Monsoon (green) and Winter (blue). Shapes signify resettled (square) and host community (circle) households with almost entirely overlapping distribution in PCA loadings space.

Our results of modelling livelihood characteristics associated with resettled and host community households highlight that increasing asset index values were positively associated with FCSs year-round (Table 1). Predominantly, households with diversified incomes, except from labor, were also the households that reported higher FCSs regardless of being resettled or not. Labor incomes were most often negatively associated with FCSs in resettled and host community households (Table 1, for model result details broken down by season see S10 File). The similarities in livelihood characteristics between resettled and host community households are further confirmed by our PCA results where resettled and host community households overlap in variable space almost entirely (Fig 6).

**Table 1:** Variables with significant associations to FCSs for resettled and host community households.

Variable Group	Variable	Resettled	Host Community
Assets and Resources	Assets	+	+
	Poultry Owned	+	+
	Cattle Owned	+	+
	Self-reported forest access for all uses	+	+
	Forest foods consumed (from market)	+	NE
	Kitchen Gardens	+	NE
	Time since resettlement	+	NE
	Number of relatives in the area	+	NE
	Land owned	NE	+
	Winter Crop	NE	+
	Winter crop % sale	NE	+
Coping strategies	CSI	+	+ (-ve in Summer)
	Children sent to school to eat a meal	NE	-
	Monsoon crop % consumed	NE	-
Types of Income Diversification	Agriculture + labor income	+/-	-
	Labor only income	-	-
	Agriculture + poultry income	+	+
	Agriculture + stable job income	+	NE
	Stable Job income	NE	+
	Tendu work effort	+	-
	Agriculture + cattle income	-	NE
	Cattle income only	-	NE
Remoteness	Distance to market	-	NE
	Distance to city	NE	-

Linear mixed model results with random effects of *tehsil* and origin village as categorical variables. ‘**+**’ and ‘**–**’ are used to signify positive and negative associations respectively (NE = no significant effect). For estimates and other details please see–S10 File.

Apart from the above livelihood associations with FCSs, resettled households supplement market bought foods (>90% from market across all surveys) with self-provisioned foods (non-forest local seasonal greens from village areas and kitchen gardens) while host community households engage in winter cropping to attain similar FCSs (Table 1). Owning cattle is positively associated with FCS for all households while incomes from cattle are negatively associated for resettled households. Distances to markets and built up areas were negatively associated with FCSs (for resettled households and their host community neighbors respectively). Finally, resettled households also exhibit a temporal aspect with months since resettlement being positively associated with FCSs (Table 1 and see S10 File for seasonality of variables in models).

## Discussion

Our study contributes to the growing quantitative assessment of resettlement on human well-being measurements, tying together conservation and social goals [16, 65, 66]. The future of poverty reduction and conservation depends on co-managed conservation outside of protected areas and the ethical inclusion of peoples’ needs as the forefront of policies within conservation landscapes [14, 32–34]. The study focusses on a human-dominated landscape surrounding a protected area, in contrast to other literature in which the landscape is less densely populated and less integrated with markets [31, 67, 68]. In regions of the world with acute land competition, such as India, assessments of conservation action will aid in developing guidelines for resettlement policies. Resettlement of local communities is tied to the origins of the first formally declared protected areas for wildlife and historically been mired in justified criticisms for the top-down approach with little consideration for local communities’ well-being [9–11]. In the last few decades, authorities have revised historically oppressive conservation polices in response to extensive critique to better reflect the intertwined nature of protected area management and the well-being of local communities [36, 69]. However, in the case of resettlement, many areas of study still require greater transparency and collaborations [12, 13]. The study of resettlement is integral to the conservation-development field, especially in protected area landscapes [7, 12, 44, 66, 70]. Our study forms a data rich case study within landscape trade-off frameworks to reach multiple goals for people and wildlife (see—[39, 68]). Finally, our study provides a measurable, meaningful and replicable way to understand post resettlement dynamics across these landscapes where people and conservation are intricately connected. We discuss in detail the contribution of our study to the overall understanding of people living around protected areas.

### Incorporating food security as a critical dimension of social outcomes from resettlement

Our study finds widespread food insecurity for all households. However, the current state of food insecurity around KNP is not acute with very few reports of hunger and coping strategies associated with higher food access. Food insecurity is largely due to the economic inability to access high nutrient food groups (Table 1, [69]). Our finding mirrors global patterns inadequate economic access to nutritious foods for more than 1.6 billion people [71]. Around KNP, diets largely comprise of cereals, tubers and pulses. Households with acceptable FCSs in our study include vegetables only five days of the week in their diets. In our exploration of livelihood characteristics associated with the prevalent food insecurity, we highlight the lack of stable incomes as an important consideration. Most households rely on a single rain-fed cropping system to provide the mainstay annual household income, and with unpredictable monsoon rainfall the reliance on irrigation or other incomes becomes heightened. Our study highlights the importance of local context in post-resettlement studies to ensure human development alongside conservation practice [20, 21]. Researchers have extensively explored the impacts of other conservation actions on local human-communities as well as landscape level trade-offs to manage wildlife and development goals [36, 69]. Quantitative studies on the impacts of resettlement are now possible, in part due to documentation of resettlement [16, 41, 65]. Researchers can begin to explore how resettlement as a particular conservation practice affects people and future management of landscapes. The surveyed villages around KNP require attention for food security and livelihood options for future stability and increased quality of life, especially in monsoon when food insecurity increases.

The reasons for monsoon being a difficult season for most households are twofold–the first is the time costs on households in the main growing season for labor intensive paddy crop and the second is the logistical difficulty in getting to markets during monsoon rains. Moreover, as the KNP remains closed for tourism during monsoon, any incomes from eco-tourism are non-existent during this time, perhaps affecting the local economies where our surveys were conducted. In Baihar *tehsil* (administrative block) there were seasonally changing differences in resettled and host community household FCSs and CSIs but these require further study to understand the underlying mechanisms (S7 File). Livelihoods in Baihar *tehsil* are likely more influenced by a few towns and tourism infrastructure in close proximity to each other and our surveyed villages. Established local communities might be more adapted to the dynamics of urbanizing livelihoods than newly resettled households that employ mild coping strategies more frequently to attain similar FCSs in these areas.

Apart from increasing overall economic wealth, resettled households rely on local resources (tendu trade, gathering local village area greens and kitchen gardens) while host community households predominantly rely on winter cropping to end up with similar FCSs (Table 1). Our results suggest that host communities are established agrarian communities within which some households have been able to avail of irrigation facilities required for winter cropping, whereas resettled households have not ventured into winter cropping for income diversification. Longer studies, or studies after a few years of resettlement, are required to understand why resettled households do not winter crop for income diversification, especially given their chance to overcome high initial investments with the resettlement monetary compensation and similar amounts of land owned as well as other assets (S1 File). The positive association of more relatives in the area for resettled households is also explanatory as a better social network to aid in coping strategies. We reiterate that resettled households were agrarian even when living within KNP and likely incurred higher losses from human-wildlife conflict as well as the lack of irrigation facilities or modernized farm machinery. Culturally, resettled households and their host community neighbors were from the same tribes prevalent in the region with similar beliefs and agrarian livelihoods. We document these nuances from our analyses so that the results of our study can aid in tailored interventions to alleviate food insecurity more accurately and therefore more efficiently. Our findings suggest that coping strategies are the path to avoid hunger in the landscape and therefore are positively associated with FCSs in our model results, this is reinforced when we consider that most households only use mild coping strategies and sparingly. We reiterate that our results of livelihood characteristics that are associated with FCSs are not causal or predictive but more descriptive of existing livelihoods and food insecurity in the KNP landscape.

These differences aside, both resettled and host community households with increasing assets and diversified incomes showed positive associations with FCSs. Households with incomes from poultry and agriculture had comparable positive associations with FCSs as salaried jobs. We suggest that gains from poultry farming might be an avenue for on-the-ground interventions that aid alleviation of poverty as well as food insecurity [72, 73]. We also suggest that local authorities and Non-Governmental Organizations (NGOs), with programs on livelihoods and well-being, explore avenues to increase monsoon incomes annually by utilizing idle tourism resources. Finally, we emphasize exploring low cost (economic and time) interventions such as kitchen gardens and maintaining commons to allow poor and borderline FCSs households to access fresh vegetables more frequently without market reliance. Villages within the KNP landscape might also consider maintaining commons as forest wood lots for fuelwood, grazing lands and other uses aligning with larger conservation goals in the region for habitat connectivity and forest restoration targets.

### Quantifying food security of people living around protected areas where people are connected to markets

The key objective of our study was to assess post-resettlement food security of resettled compared to their host community households at the new settlement location in the KNP landscape. Our findings suggest that the current resettlement does not constrain resettled households and that they are comparable to host community households in terms of livelihood opportunities, physical food availability and food access. Approximately 20% of our surveyed households are within the KNP buffer. Households within the administrative buffer of KNP are more similar to households outside the KNP than those previously living within the KNP core in terms of economic activities, access to the road network in the KNP landscape and commercial livelihoods (personal observation). The multiuse buffer around KNP is predominantly agricultural land with more than 260 existing villages. Resettled households join existing villages outside of KNP and comprise a minor proportion of the total village population [63].

To increase overall wealth, an obvious but difficult application of our results would be to generate more opportunities for salaried jobs and make agricultural practices more profitable. Such an obvious result seems a moot point to discuss but we do so to ensure our results are not seen as a contrast of two separate livelihoods–resettled households with forest livelihoods separated from rural host community households with more wealth driven livelihoods. Our results confirm that the reality is one where rural livelihoods predominantly feature wealth accruement and market bought foods regardless of being resettled and host community households (Table 1). Our study findings contrast with multiple studies of food security, in less populated conservation areas, where people rely directly on forests for food or where increased incomes do not track with increased food security [31, 74, 75]. We also found that different sources of household income are associated differently with FCSs. A commonly adopted additional livelihood, daily wage labor is associated with households that have poor FCSs around KNP. We caution readers that our results do not suggest that labor incomes cause food insecurity. Instead we point out that households with lower FCSs are also households that are involved in labor for incomes.

Owning more cattle suggests that households have means to increase assets which are in turn associated with higher FCSs. Cattle are culturally important, most often as work animals and easily traded assets in times of distress. Cattle for dairy incomes are rare in the KNP landscape. Incomes from cattle might be occasional (distress sale) or low (dairy farming practices). Similar findings about the complicated relationship between livelihoods and cattle owning have been found in Ghana [35]. We also found that self-reported forest access has a positive association with FCSs in our study but in our surveys there is little evidence of direct reliance on forests for food (S6 File). A possible explanation is that access to forests provide for supportive uses to food security by providing spaces to graze cattle, gather fuelwood, seasonal Tendu incomes and collect seasonal foods that are market sold [68, 76]. Our results about using natural resources and forests for food security contribute to the growing body of research on linkages between food and forests (see—[21, 24, 29]), adding empirical findings from KNP—a human-dominated protected area landscape. Access to forest is complicated in India, as in many places across the planet, with national policies and state control that lead to vulnerable peoples with few rights, low levels of individual or collective agency, and weak governance over their resources [77–79]. While our study does not include analyses on this aspect, in the central India region there has been recent evidence of how elite capture and modified state control continue to impede co-management of resources by communities surrounding protected areas [80, 81].

Food security for resettled households, and their host community neighbors, in the Kanha National Park landscape will be best achieved by integrating the poorer households into the economy with more opportunities, such as steady jobs and poultry farming, for higher incomes and seasonal stability. Managers for resettlement and rural development around KNP, and in other human-dominated conservation landscapes, might consider the importance of training for employment with steady incomes in future interventions. At the national policy level, the NTCA can use the methods from our study to explore links between livelihoods and social goals in resettlement from tiger reserves across India that vary ecologically, geographically and culturally. While this study concluded that steady incomes are highly relevant for food security in the KNP landscape, other landscapes around tiger reserves might indicate the need for access to forests or efforts to improve food availability.

Our study focuses on a short time-frame after resettlement to look at immediate impacts on food security as a metric of human well-being. Longer studies or studies after a decade of resettlement might provide further insight into how compensations under the current guidelines allow resettled households to integrate into their new locations. Furthermore, studies that include metrics of well-being that are difficult to quantify are important to understand social impacts on people living around protected areas [41, 65]. Studies have found that understanding local contexts aids researchers in a more nuanced exploration of the impacts of conservation practice even when hard to quantify [18, 19]. Finally, our data was not collected to measure the strength of local institutions which are important to study and might lead to different outcomes for people and wildlife alike.

## Conclusions

We conclude that there is low level of food insecurity in the KNP landscape that is largely driven by low and unstable household incomes. Resettled households are comparable to their host community neighbors in food availability (distance to markets, roads and availability of forests). We also found that resettled households are comparable to their host community neighbors in food security (FCSs and CSIs) and that households with higher food security had more assets as well as more income sources. Resettled households have more seasonal income sources and supplement foods locally (kitchen gardens) while their more established host community neighbors rely on winter cropping incomes to attain similar food security. Our results suggest that increased opportunities for diversifying non-labor incomes could be effective to alleviate food insecurity for both resettled and host community households in the KNP landscape. This result reinforces multiple studies that highlight livelihood diversification as a means for alleviating household food insecurity [15, 66]. Our results for the KNP landscape suggest that access to forest resources is less critical for food security than the ability to purchase food from local markets. While other studies indicate the importance of access to forests for food security in other landscapes [29, 31, 68], we posit that the role of forests in food security is highly dependent on the local context and integration of households with markets. A general conclusion about the relevance of forests for food security cannot be applied to all landscapes.

## Supporting information

S1 File Propensity score matching for balance testing in our data. (PDF)

S2 File Food security metrics. (PDF)

S3 File Distance from KNP and forest cover. (PDF)

S4 File Variables used in PCA (Fig 3C). (PDF)

S5 File Market data. (PDF)

S6 File Household food sources. (PDF)

S7 File CSI by tehsil and season. (PDF)

S8 File Variables used in models (code and correlation matrix). (PDF)

S9 File Random forest output. (PDF)

S10 File Model details–significance of variable and effect sizes. (PDF)
