# Supplementary material for: Food security and livelihoods of post-resettlement households around Kanha National Park
Source: PLoS One. 2020 Dec 28;15(12):e0243825. doi: 10.1371/journal.pone.0243825 (PMC7769436; doi:10.1371/journal.pone.0243825)
Supplement: S1 File — (PDF) [file pone.0243825.s001.pdf]

1. Propensity score matching (without replacement) used for balance testing in our data.

We used propensity matching to check for balance in our study groups – resettled and host community households. We used a strict one-to-one matching setting to ensure we were being robust in our balance check. Since we found most resettled households were matched to one of our host community households, we considered the groups similar enough to use all our data in all further analyses. We performed propensity scoring to check balance after conducting the surveys since we had no data on which to base the scoring prior to the survey.

Table 1: We report the means and standard deviations of the characteristics in the variables used for propensity scoring to emphasize that resettled and host community households are comparable in our study.

Variable	Mean Resettled (SD)	Mean Non-resettled (SD)
Land Owned	2.0 (1.6)	2.5 (4.8)
Assets	5.2 (4.3)	5.5 (4.7)
Shock Incidence	0.02 (0.1)	0.03 (0.1)
Winter Crop Types	0.4 (0.7)	0.5 (0.8)
Winter Crop (y/n)	Categorical	
Years of education	5.8 (4.7)	7.2 (5.2)
Number of adults	3.6 (1.8)	3.8 (1.8)
Number of children	1.9 (1.4)	1.9 (1.4)
Number of relatives	1.5 (4.0)	2.5 (6.0)
Tribe & caste	Categorical	
% of wheat crop sold	0.4 (3.9)	0.8 (6.0)

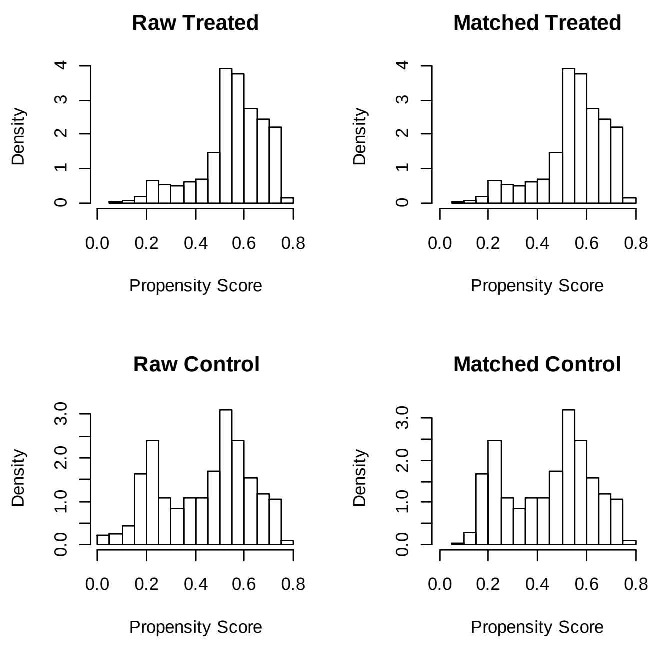

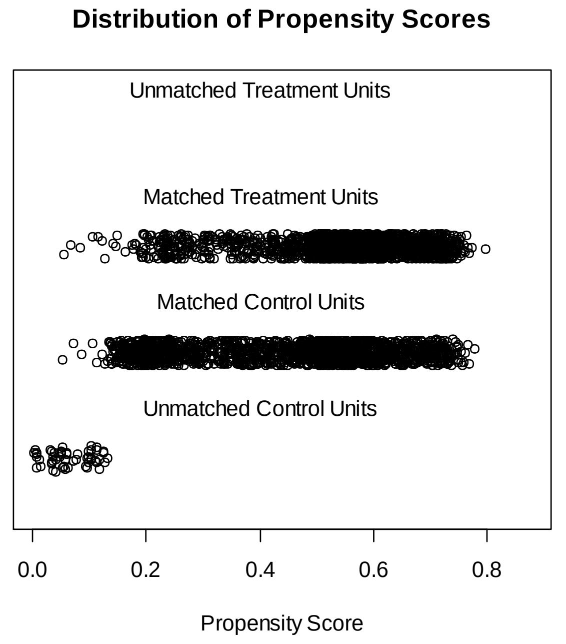

Output histograms and jitter plots show balance in study in terms of comparing resettled (treatment) to host community (control) households with the heavy penalization in one-to-one matching.
