# Supplementary material for: Food security and livelihoods of post-resettlement households around Kanha National Park
Source: PLoS One. 2020 Dec 28;15(12):e0243825. doi: 10.1371/journal.pone.0243825 (PMC7769436; doi:10.1371/journal.pone.0243825)
Supplement: S2 File — (PDF) [file pone.0243825.s002.pdf]

2. Food security metrics:

We used three metrics in our study to measure household access to food – the Food Consumption Score (FCS), the Coping Strategy Index (CSI) and the Household Hunger Scale (HHS).

“The Food Consumption Score (FCS) is an index that was developed by the World Food Programme (WFP) in 1996. The FCS aggregates household-level data on the diversity and frequency of food groups consumed over the previous seven days, which is then weighted according to the relative nutritional value of the consumed food groups. For instance, food groups containing nutritionally dense foods, such as animal products, are given greater weight than those containing less nutritionally dense foods, such as tubers. Based on this score, a household’s food consumption can be further classified into one of three categories: poor, borderline, or acceptable.” (INDDEX Project, 2018; World Food Programme, 2008)

“The Coping Strategies Index (CSI) is an indicator of household food security that is relatively simple and quick to use, straightforward to understand, and correlates well with more complex measures of food security. A series of questions about how households manage to cope with a shortfall in food for consumption results in a simple numeric score. In its simplest form, monitoring changes in the CSI score indicates whether household food security status is declining or improving. The CSI is based on the many possible answers to one single question: “What do you do when you don’t have adequate food, and don’t have the money to buy food?” The CSI requires some “up-front” work to ensure that all the potential answers to this question

are known, and that it is adequately adapted to the local situation.” (Maxwell and Caldwell, 2008)

“The approach used by the HHS is based on the idea that the experience of household food deprivation causes predictable reactions that can be captured through a survey and summarized in a scale. This approach, sometimes referred to as an “experiential” or “perception-based” method of collecting data, was first popularized in the mid-1990s, when the United States Department of Agriculture (USDA) adopted the approach for routine measurement of household food insecurity in the United States.” (Ballard et al., 2011)

In addition to the above explanations, we add the following study specific information:

The HHS only pertains to dire food insecurity and asks for a recall period of 30 days / 4 weeks. We use is with our other metrics to catch high food insecurity in the households that otherwise respond to our queries about food with only a 7 day recall.

FCS and CSI together provides a richer interpretation of household food security status (Jones et al., 2013; Maxwell et al., 2013). For example in our study, we found that many times CSI was positively associated with FCS. In this case, we interpret our results as a household having the capacity to cope. If we found that CSI was negatively associated with FCS, we surmise that the household is trying to cope but struggling with food insecurity.

We use the FCS metric with standard thresholds for when households regularly use sugar and oil in our study (World Food Programme, 2008). Field manuals of the food access metrics used in our study stress the importance of key informant interviews and focus groups to understand how best to use the standardized metrics in a given context. Accordingly, we held key informant interviews and focus group discussions to generate a list of local names for foods as well as lesser known local foods. We also used the key informant interviews to understand the likely response to queries regarding mild to severe coping strategies. A query on begging as a coping strategy was removed from our survey instrument as begging is not a coping mechanism in this landscape (there are extremely few avenues to solicit food by begging unlike in large cities) and the query was judged as highly culturally insensitive. We conducted focus groups discussions with three village families from non-survey villages. Key informants ranged from villagers, drivers, managers and local people who lived in non-survey areas including a town family. Key informants were available and helpful throughout the duration of our field surveys but we gathered most local information during two pilot studies prior to these surveys.
