# Supplementary material for: Food security and livelihoods of post-resettlement households around Kanha National Park
Source: PLoS One. 2020 Dec 28;15(12):e0243825. doi: 10.1371/journal.pone.0243825 (PMC7769436; doi:10.1371/journal.pone.0243825)
Supplement: S3 File — (PDF) [file pone.0243825.s003.pdf]

3. Distance from KNP and forest cover

(a)

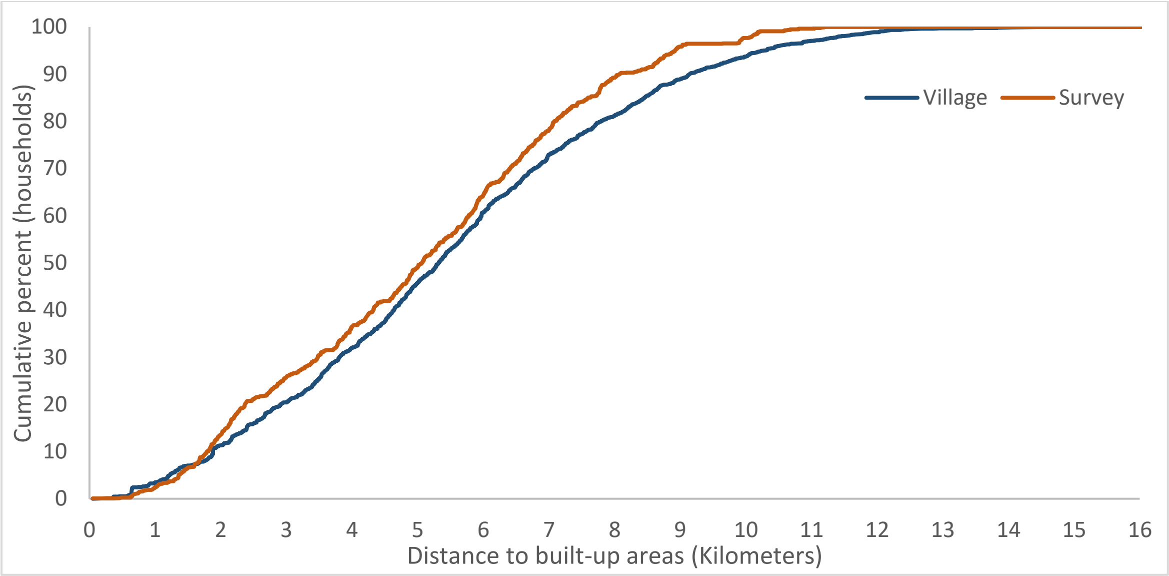

(b)

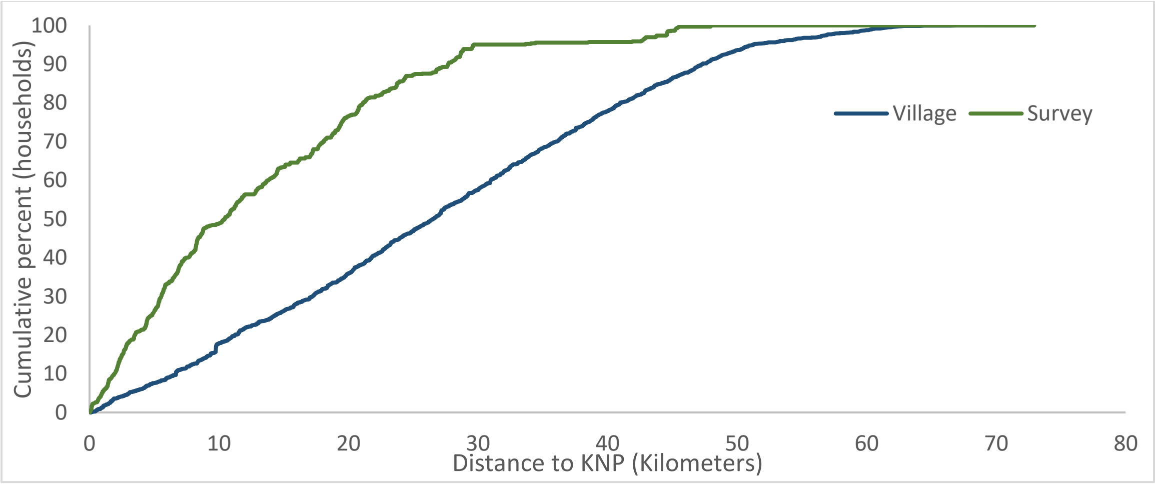

(c)

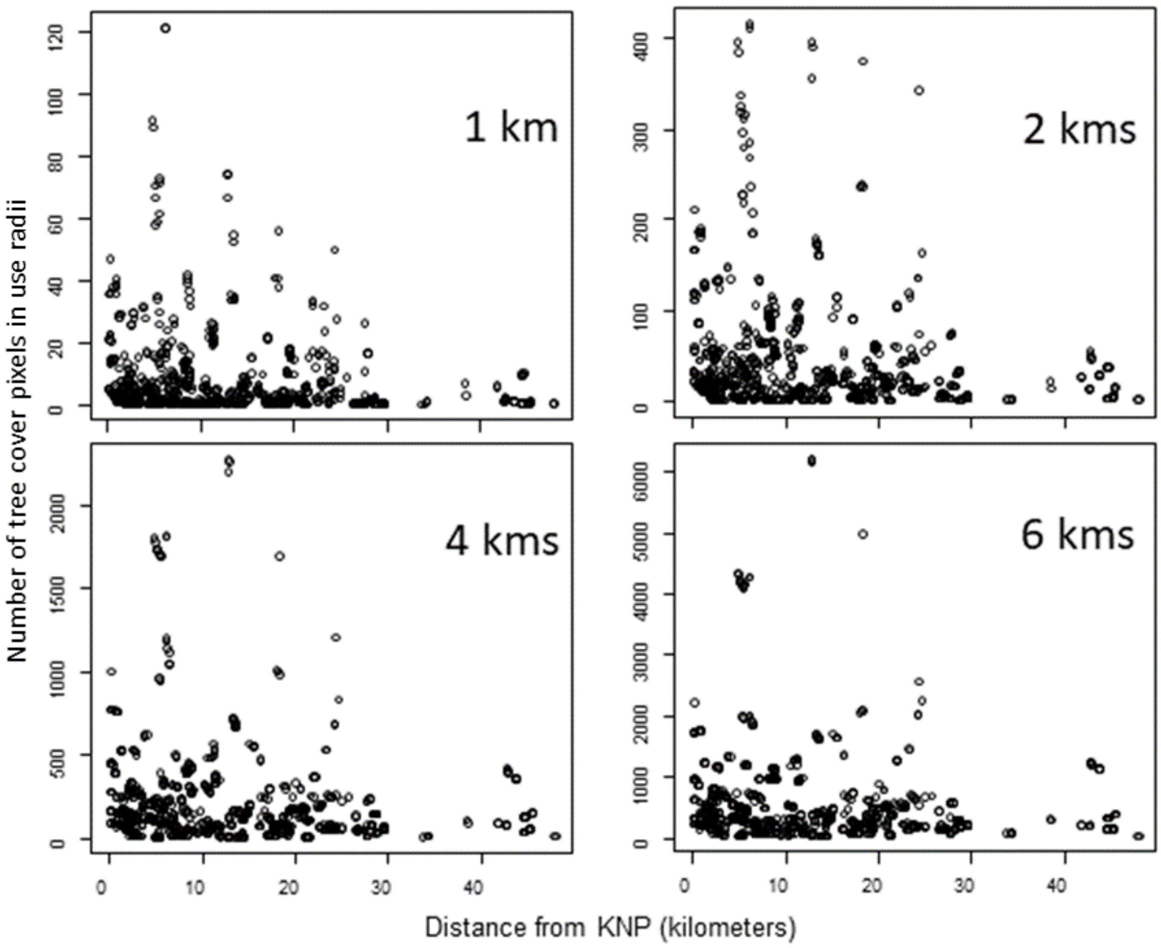

Our survey data is representative of the larger KNP landscape human populations in terms of distance from built-up areas (a). We find that our sample is less representative of the larger landscape when considering distance to KNP perimeter (b) and we show that although tree cover decreases with increasing distance from KNP most households have similar mean tree cover in our sample (c).
