# Supplementary material for: Food security and livelihoods of post-resettlement households around Kanha National Park
Source: PLoS One. 2020 Dec 28;15(12):e0243825. doi: 10.1371/journal.pone.0243825 (PMC7769436; doi:10.1371/journal.pone.0243825)

## 4. VARIABLES USE IN PCA (FIG 3c)

| Variable as per (Census 2011) | Mean for surveyed villages | Mean for all villages in study extent | P-value |
|---|---|---|---|
| total village area (area) | 369.47 | 335.47 | 0.18 |
| forest land (forest) | 63.67 | 66.04 | 0.83 |
| unirrigated land (unirr) | 165.79 | 204.93 | 0.37 |
| presence of national highway (NH=1 when present at village, 2 when not) | 1.94 | 1.92 | 0.46 |
| presence of state highway (SH=1 when present at village, 2 when not) | 1.91 | 1.85 | 0.04 |
| distance to nearest town (towndist) | 22.75 | 20.82 | 0.36 |

**Density graphs of variables used in PCA showing considerable overlap (green) between surveyed villages (blue) and all villages in study extent (yellow) (Fig. 3(c)).** The variables we used to run this PCA were: total village area (area), forest land (forest), unirrigated land (unirr), presence of national highway (NH=1), presence of state highway (SH=1) and distance to nearest town (towndist).

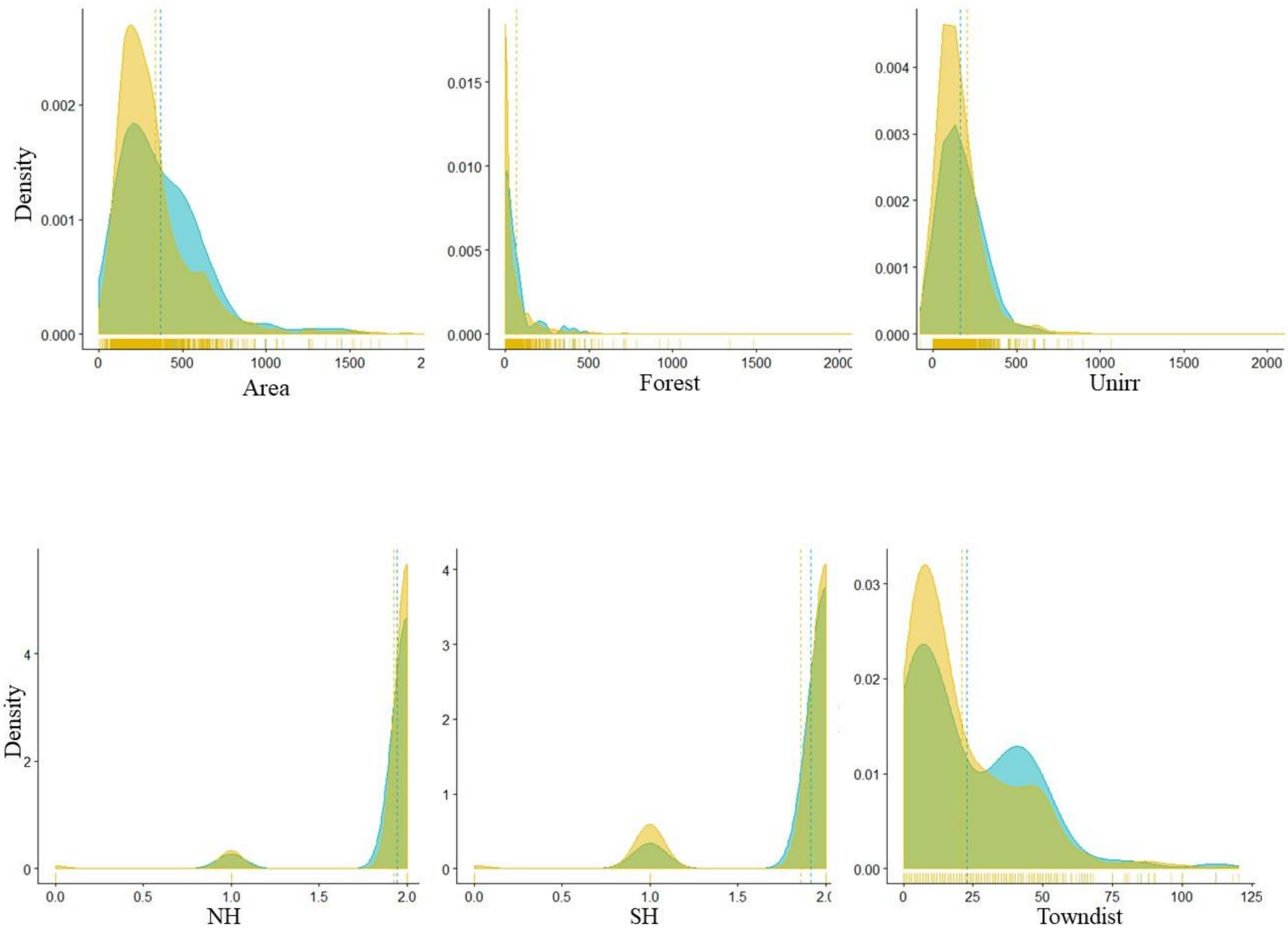

Supplement: S4 File — (PDF) [file pone.0243825.s004.pdf]
