# Supplementary material for: Food security and livelihoods of post-resettlement households around Kanha National Park
Source: PLoS One. 2020 Dec 28;15(12):e0243825. doi: 10.1371/journal.pone.0243825 (PMC7769436; doi:10.1371/journal.pone.0243825)
Supplement: S5 File — (PDF) [file pone.0243825.s005.pdf]

5. Market data across the study region

Food Group	Gadhi Market (INR)	Baihar Market (INR)	Mandai Market (INR)	Malajkhand Market (INR)	Bodla Market (INR)
Cereals					
Tubers	20	20	20	20	17
Pulses	80	63	89	84	83
Red Meat	340	345	350	340	340
Chicken	147	140	145	145	140
Eggs				60	55
Fish	176	130	135	135	135
Dry Fish	120				
Vegetables	32	33	27	27	28
Pirahi Mushrooms			275	365	365
Putpura Mushrooms	80	80	80	130	145
Fruit				63	
Snacks	y	y	y	y	y
Oil	85	95	113	97	97
Sugar	32	40	37	37	37
Salt	17			19	20
