# Supplementary material for: Food security and livelihoods of post-resettlement households around Kanha National Park
Source: PLoS One. 2020 Dec 28;15(12):e0243825. doi: 10.1371/journal.pone.0243825 (PMC7769436; doi:10.1371/journal.pone.0243825)
Supplement: S6 File — (PDF) [file pone.0243825.s006.pdf]

6. Breakdown of households that accessed foods via market purchase, own production and foraging across all seasons (recorded as primary and secondary sources in FCSs measurements)

Summer primary food sources

	Market Purchased	Own Production	Foraged	Total Households Reporting Access Of Food
Maize	78	122	0	202
Rice	443	877	0	1332
Wheat	429	94	0	571
Tuber	1214	39	0	1253
Peanuts	28	3	0	31
Pulses	1101	113	0	1216
Fish	334	3	13	350
Dry Fish	29	1	1	31
Red Meat	23	3	0	26
White Meat	143	30	0	174
Oil	1288	27	0	1315
Eggs	104	30	0	135
Milk In Tea	86	63	0	150
Dairy	15	44	0	60
Vegetables	1283	24	0	1308
Fruits	117	7	0	125
Wild Meat	2	0	0	2
Sweets	659	2	0	663

Summer secondary food sources

	Market Purchased	Own Production	Foraged	Total Households Reporting Access Of Food
Maize	24	6	0	34
Rice	576	79	0	752
Wheat	88	21	0	123
Tuber	26	20	1	47
Peanuts	4	0	0	4
Pulses	56	39	0	99
Fish	2	0	1	3
Dry Fish	0	0	0	0
Red Meat	0	0	0	1
White Meat	0	1	0	3
Oil	16	9	0	28
Eggs	2	2	0	4
Milk In Tea	1	1	0	7
Dairy	0	1	0	1
Vegetables	17	27	0	45
Fruits	1	1	0	3
Wild Meat	0	0	0	0
Sweets	0	0	0	0

Monsoon primary food sources

	Market Purchased	Own Production	Foraged	Total Households Reporting Access Of Food
Maize	25	43	0	71
Rice	633	428	0	1064
Wheat	323	34	0	357
Tuber	876	13	0	889
Peanuts	23	1	0	25
Pulses	901	70	0	973
Fish	217	12	7	238
Dry Fish	18	0	0	18
Red Meat	8	1	0	9
White Meat	169	19	0	190
Oil	993	10	0	1003
Eggs	54	1	0	55
Milk In Tea	15	49	0	66
Dairy	5	29	0	36
Vegetables	995	26	3	1025
Fruits	62	6	1	69
Wild Meat	1	0	2	3
Sweets	706	1	0	707

Monsoon secondary food sources

	Market Purchased	Own Production	Foraged	Total Households Reporting Access Of Food
Maize	1	0	0	2
Rice	134	96	0	244
Wheat	2	2	0	6
Tuber	6	3	1	13
Peanuts	0	0	0	0
Pulses	11	4	0	15
Fish	2	0	0	3
Dry Fish	0	0	0	0
Red Meat	0	0	0	0
White Meat	0	0	0	1
Oil	4	0	0	10
Eggs	0	0	0	0
Milk In Tea	0	2	0	2
Dairy	0	0	0	1
Vegetables	11	12	7	36
Fruits	0	0	0	0
Wild Meat	0	0	0	0
Sweets	2	0	0	3

Winter primary food sources

	Market Purchased	Own Production	Foraged	Total Households Reporting Access Of Food
Maize	28	181	0	210
Rice	279	841	0	1120
Wheat	426	41	0	467
Tuber	998	59	0	1060
Peanuts	52	1	0	53
Pulses	1003	46	0	1049
Fish	353	8	1	362
Dry Fish	25	2	0	27
Red Meat	7	1	0	10
White Meat	235	18	0	254
Oil	1083	29	0	1112
Eggs	77	15	0	92
Milk In Tea	18	36	0	54
Dairy	5	39	0	44
Vegetables	1019	96	0	1115
Fruits	36	15	0	53
Wild Meat	2	0	0	2
Sweets	462	0	0	462

Winter secondary food sources

	Market Purchased	Own Production	Foraged	Total Households Reporting Access Of Food
Maize	6	2	0	8
Rice	338	58	0	399
Wheat	30	2	0	34
Tuber	34	18	0	54
Peanuts	0	0	0	0
Pulses	29	7	0	38
Fish	2	0	0	2
Dry Fish	1	0	0	1
Red Meat	0	0	0	2
White Meat	3	2	0	5
Oil	22	6	0	28
Eggs	1	2	0	3
Milk In Tea	2	2	0	4
Dairy	0	1	0	1
Vegetables	80	145	0	227
Fruits	3	1	0	4
Wild Meat	0	0	0	0
Sweets	2	0	0	4
