# Supplementary material for: Food security and livelihoods of post-resettlement households around Kanha National Park
Source: PLoS One. 2020 Dec 28;15(12):e0243825. doi: 10.1371/journal.pone.0243825 (PMC7769436; doi:10.1371/journal.pone.0243825)
Supplement: S7 File — (PDF) [file pone.0243825.s007.pdf]

7. CSI by *tehsil* and season

	Summer			Monsoon			Winter		
Tehsil	Resettled	Non-resettled	P-value	Resettled	Non-resettled	P-value	Resettled	Non-resettled	P-value
Baihar	2.0	2.4	0.5	6.6	2.9	**0.001**	5.4	2.5	**0.005**
Bicchiya	2.4	2.8	0.7	4.4	3.7	0.4	2.9	2.8	1.0
Birsa	1.4	1.3	0.9	4.9	4.2	0.5	3.7	3.9	0.9
Bodla	1.9	1.6	0.6	3.5	2.8	0.3	2.4	2.4	1.0
Chhuikhadan	*5.7*	*2.5*	*0.056*	2.4	0.9	0.3	1.3	1.4	0.9
Panderia	3.0	1.2	**0.045**	3.9	1.0	**0.005**	2.4	1.6	0.4
Paraswada	*1.7*	*0.5*	*0.055*	2.2	3.5	0.4	4.4	5.0	0.8
