# Supplementary material for: Food security and livelihoods of post-resettlement households around Kanha National Park
Source: PLoS One. 2020 Dec 28;15(12):e0243825. doi: 10.1371/journal.pone.0243825 (PMC7769436; doi:10.1371/journal.pone.0243825)

8(a). Variable Names as used in modelling

Variable Name	Explanation / Longer name
adn	Number of adults
asset	Asset index score
cattle	Heads of cattle
chn	Number of children
dc	Number of children suffering diarrhoea
dd	Number of days child spent suffering diarrhoea
dtc	Distance to KNP core area - Euclidean measured in QGIS
edu	Education level - highest in household
fc	Number of children suffering fever
fd	Number of days children spent suffering fever
ffc	Forest food count
fwd	Fuel Wood distance - reported by respondent on survey
fwt	Fuel Wood time taken - reported by respondent on survey
gdiv	Number of types of veg grown in garden
goat	Number of goats
hen	Number of hens
ht	House material - mud / brick+concrete / mixed
jps	Job profile: income sources (labour / agriculture / cattle / salaried job / poultry etc.)
kero	Kerosene as cooking fuel yes / no
killn	Number of livestock killed by large carnivore
land	Land owned
lpg	LPG as cooking fuel yes / no
midday	Number of days children sent to midday meals at school
mkt	Distance to market - Euclidean measured in QGIS
ov	Origin village
pcr	% crop raided by animals
pig	Number of pigs
rd	Distance to road - Euclidean measured in QGIS
reln	Number of relatives in the village
resi	Time in months since resettlement
rfn	Number of resettled families from KNP in the same village
ricec	% rice crop consumed annual
ricek	% rice crop stored annual
rices	% rice crop sold annual
riv	Distance to river - Euclidean measured in QGIS
shock	Incidence of shock event in the last year (death of family member / natural calamity)
soilf	Soil fertility perception as reported by land owner
soilw	Water retention capacity of land owned
teh	Tehsil
tendu	Number of people * number of days tendu collection effort
wheatc	% wheat crop consumed annual
wheatk	% wheat crop stored annual
wheats	% wheat crop sold annual
winterct	Winter crop type count
wppl	Number of people collecting water for household

8 (b). Variables used in models correlation matrix

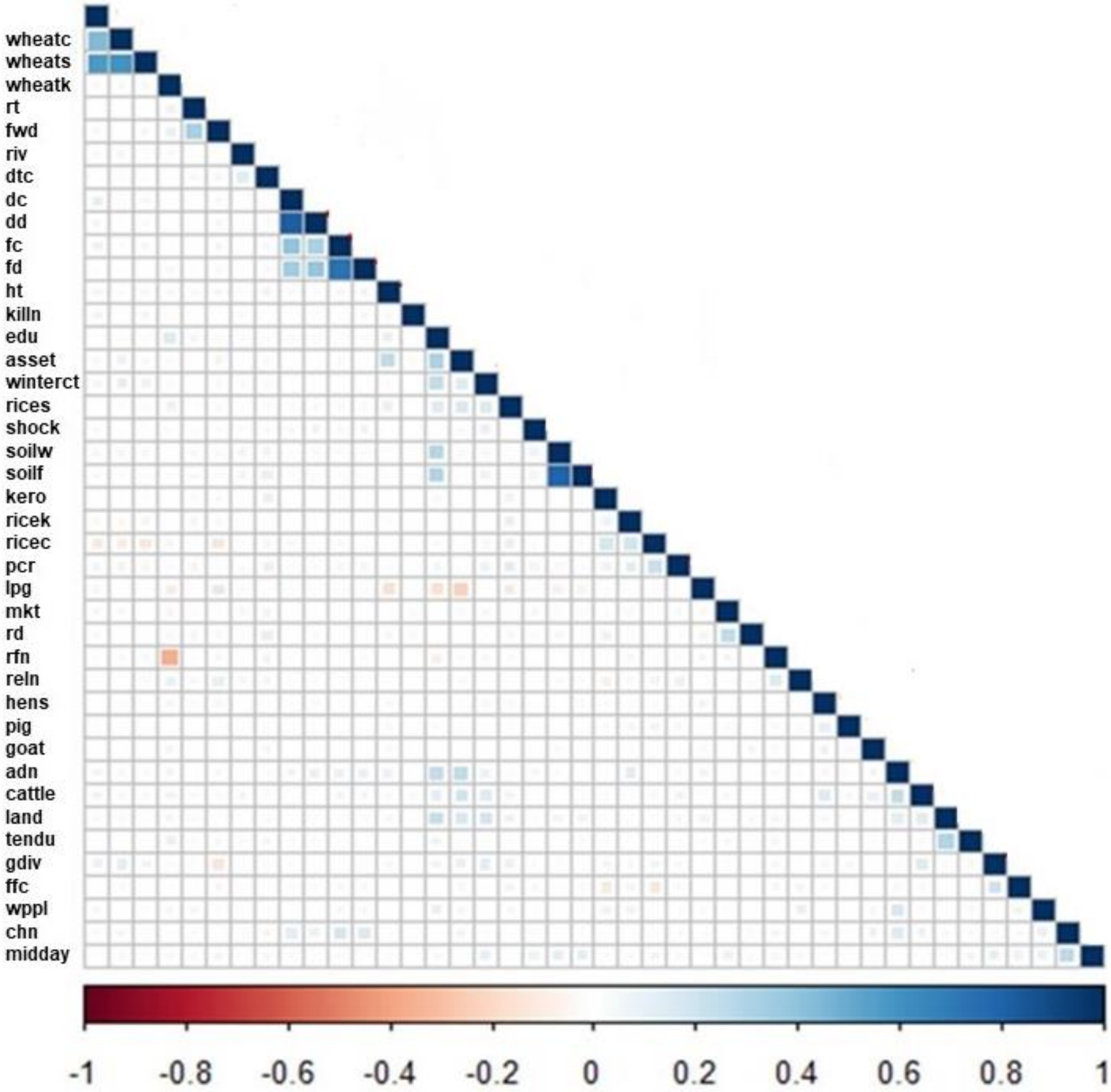

Supplement: S8 File — (PDF) [file pone.0243825.s008.pdf]
