# Supplementary material for: Food security and livelihoods of post-resettlement households around Kanha National Park
Source: PLoS One. 2020 Dec 28;15(12):e0243825. doi: 10.1371/journal.pone.0243825 (PMC7769436; doi:10.1371/journal.pone.0243825)

9. Random forest results showing important variables under various data groupings:

FCS ALL Households in our study

(Variables to do with resettlement were given a constant dummy value for host community households)

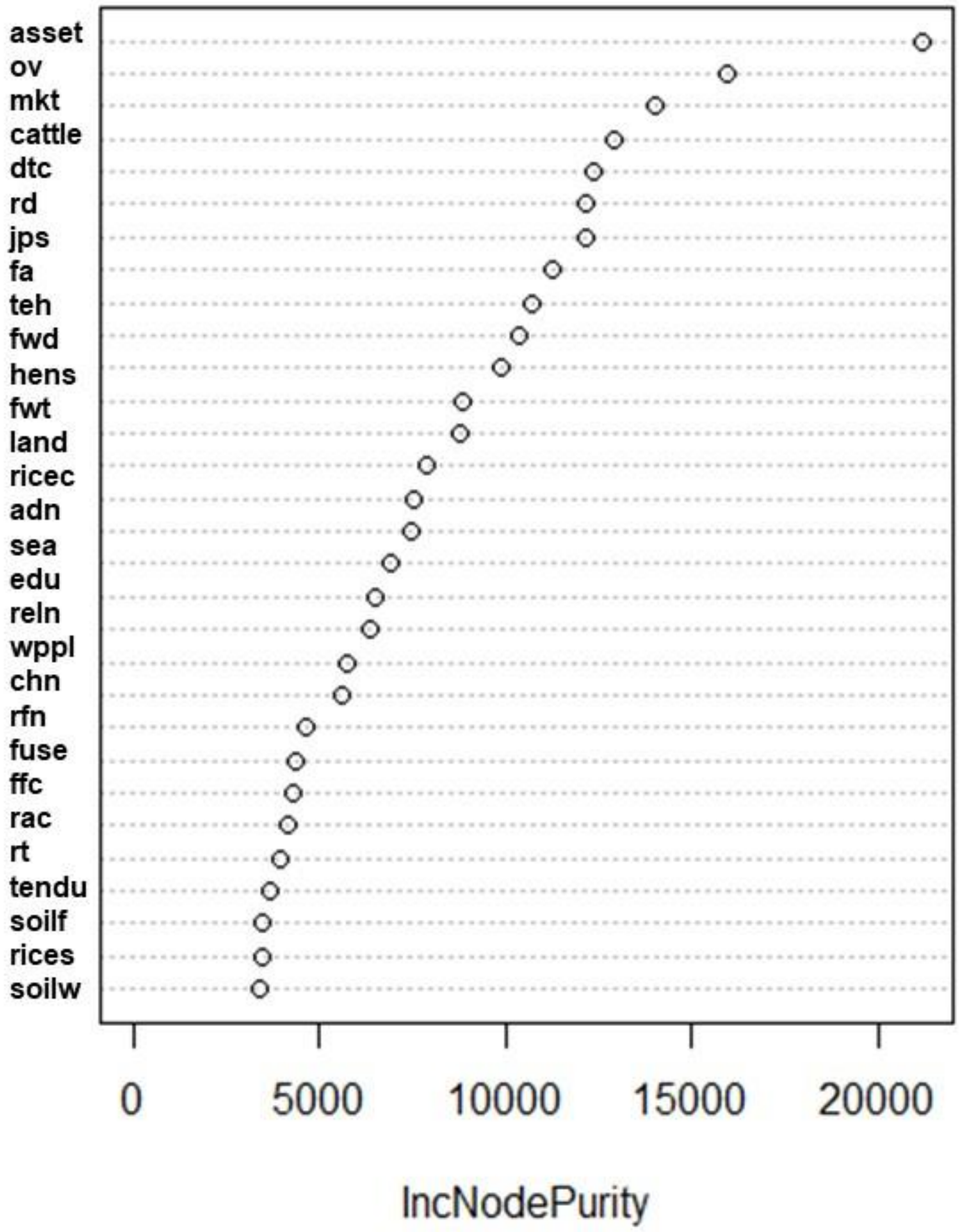

FCS Resettled with resettlement variables

(origin village, nearby resettled families, residence time)

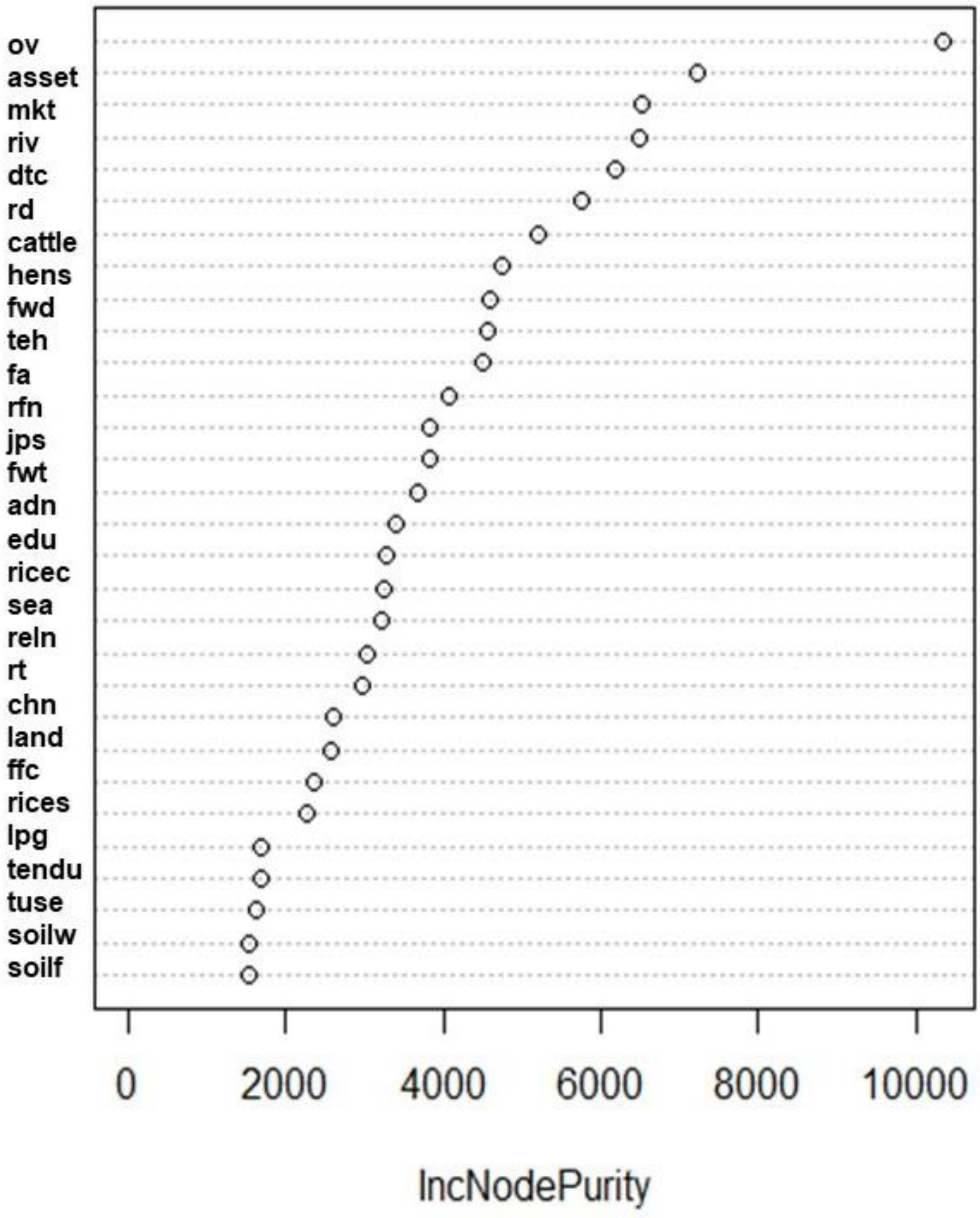

FCS Resettled with no resettlement variables (origin village, nearby resettled families, residence time)

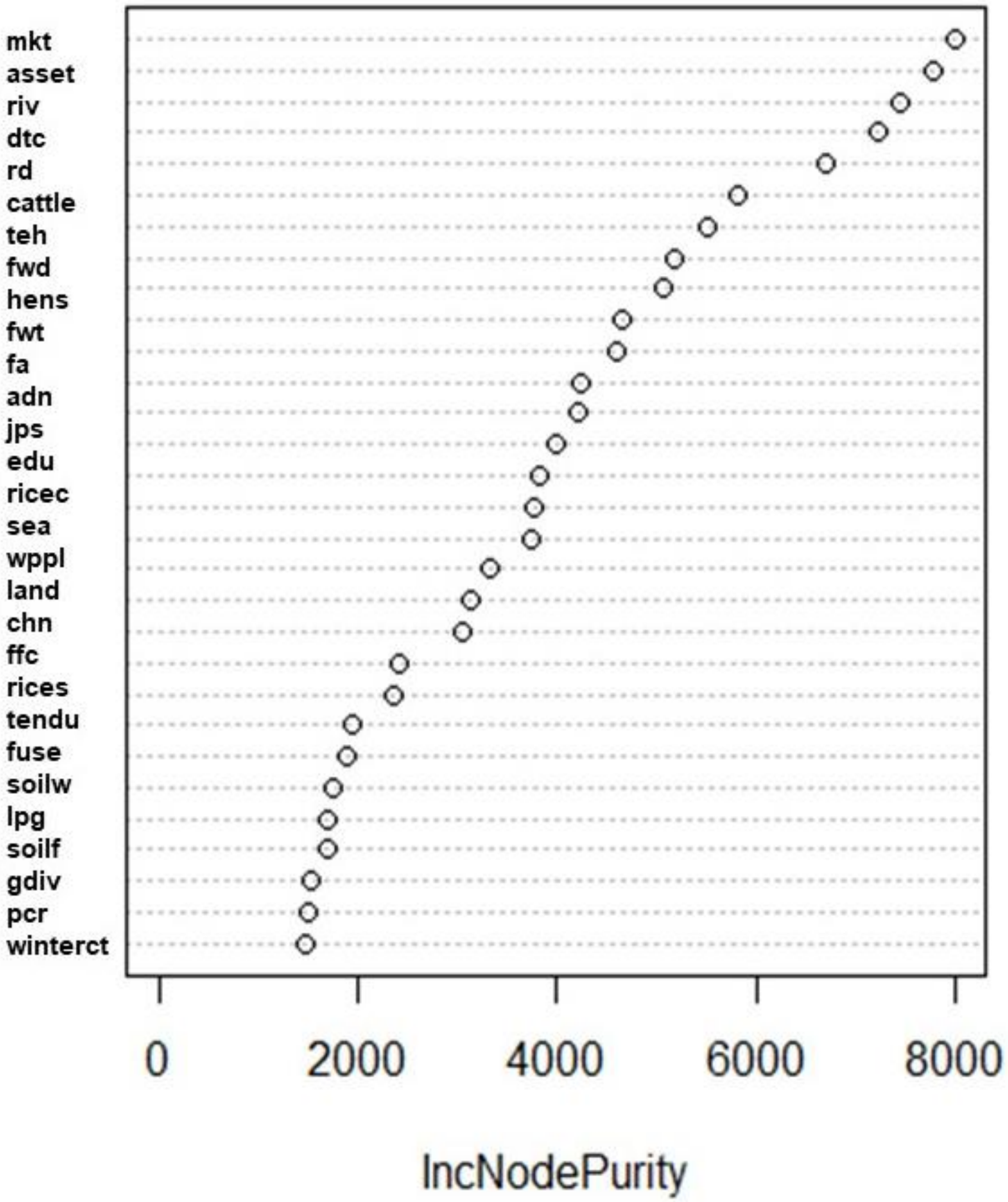

FCS for host community households only

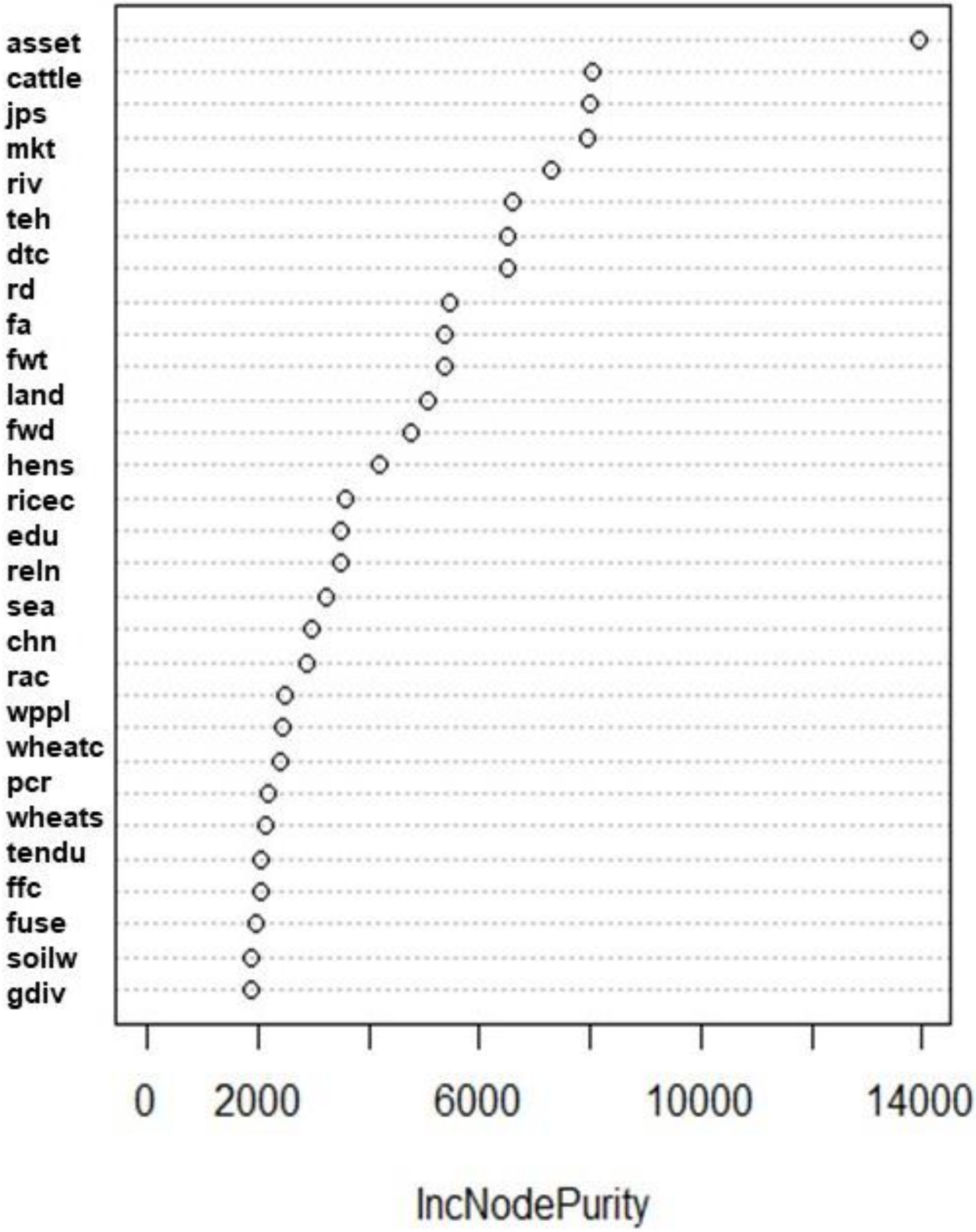

Supplement: S9 File — (PDF) [file pone.0243825.s009.pdf]
