# Supplementary material for: Food security and livelihoods of post-resettlement households around Kanha National Park
Source: PLoS One. 2020 Dec 28;15(12):e0243825. doi: 10.1371/journal.pone.0243825 (PMC7769436; doi:10.1371/journal.pone.0243825)
Supplement: S10 File — (PDF) [file pone.0243825.s010.pdf]

10. Model table with details of significance and effect sizes. Response variable FCSs across seasons.

	Summer		Monsoon		Winter	
**Marginal R square**	0.2	0.18	0.11	0.21	0.22	0.27
**Conditional R square**	0.21	0.19	0.13	0.21	0.22	0.27
**Significant Determinant Variable**	**Resettled Coefficient (SE)**	**Non-resettled coefficient (SE)**	**Resettled Coefficient (SE)**	**Non-resettled coefficient (SE)**	**Resettled Coefficient (SE)**	**Non-resettled coefficient (SE)**
FOR ALL						
Assets Index Score	0.18 *** (0.04)	0.21*** (0.04)	0.19 *** (0.05)	0.14 *** (0.04)	0.08 * (0.04)	0.23 *** (0.04)
Poultry (number owned)	0.08 * (0.04)	0.1 ** (0.04)		0.19 *** (0.04)	0.18 *** (0.05)	
Cattle (number owned)		0.11 ** (0.04)	0.16 *** (0.04)			0.18 *** (0.04)
Coping Strategies Index		-0.08 * (0.04)	0.13 *** (0.04)	0.2 *** (0.05)	0.26 *** (0.04)	0.15 *** (0.04)
Labour + agriculture incomes	0.3 *** (0.07)				-0.33 *** (0.08)	-0.26 ** (0.08)
Labour only income		-0.25 * (0.12)			-1 ** (0.32)	
Poultry + agriculture incomes	2.44 ** (0.87)					1.56 * (0.63)
Tendu Trade (people * days)	0.2 *** (0.05)			-0.11 ** (0.03)		
Forest Access self-reported: Yes	0.38 *** (0.09)	0.44 *** (0.09)		0.43 *** (0.1)	0.53 *** (0.1)	0.44 *** (0.09)

ONLY RESETTLED			
Job + agriculture incomes	1.85 *** (0.32)		
Cattle + agriculture incomes			-0.84 * (0.41)
Cattle only income			-2.15 * (0.9)
Forest Food types (count)		0.12 ** (0.04)	
Garden diversity item type (count)			0.07 * (0.04)
Time since resettlement (months)			4.55 ** (1.62)
Number of relatives in area		0.13 ** (0.04)	0.13 ** (0.05)
Distance to Market	-0.07 ‘.’ (0.04)		-0.07 ‘.’ (0.04)

ONLY NON-RESETTLED			
Job only income			1.16 ** (0.41)
Land owned (hectares)		0.17 *** (0.03)	
Count of Winter crops			0.14 *** (0.04)
% Wheat sold		0.11 *** (0.03)	
% Rice crop consumed			-0.14 *** (0.04)
Midday meals for children at school (days/week)	-0.1 ** (0.03)		
Distance to built-up area		-0.1 * (0.04)	
